# Biology-aware scaling of microalgal biofuels: bioprocess constraints and data-centric digital twins

**DOI:** 10.3389/fmicb.2026.1899560

**Published:** 2026-07-29

**Authors:** Hans Christian Correa-Aguado, Gloria Viviana Cerrillo-Rojas, Mario Alberto Arzate-Cárdenas, Ramón Jaramillo-Martínez, Jorge I. Galván-Tejada, Carlos E. Galván-Tejada, Antonio García-Domínguez, Irma González-Curiel, Karina Trejo-Vázquez

**Affiliations:** 1 Instituto Politécnico Nacional, Unidad Profesional Interdisciplinaria de Ingeniería Campus Zacatecas (UPIIZ), Zacatecas, México; 2 Laboratorio de Inmunotoxicología, Unidad Académica de Ciencias Químicas, Universidad Autónoma de Zacatecas, Zacatecas, México; 3 Departamento de Química, Centro de Ciencias Básicas, Universidad Autónoma de Aguascalientes, Aguascalientes, México; 4 Centro de Investigación e Innovación Biomédica e Informática, Unidad Académica de Ingeniería Eléctrica, Universidad Autónoma de Zacatecas, Zacatecas, México

**Keywords:** biology-aware modeling, circular biorefineries, digital bioprocessing, hybrid modeling, microalgal cultivation, process observability, soft sensors

## Abstract

Microalgal biofuels remain a promising route for renewable fuel production, carbon utilization, wastewater valorization, and the development of a circular bioeconomy. However, their industrial deployment is still limited by the difficulty of translating laboratory performance into robust, economically viable, and environmentally sustainable large-scale systems. This review presents a biology-aware and data-centric perspective that connects biological, engineering, operational, techno-economic, and computational dimensions to address industrial microalgal biofuel scale-up. It focuses on process observability, soft sensors, and the data requirements needed to transform microalgal cultivation into a measurable, modellable, and controllable bioprocess. It further evaluates artificial intelligence, hybrid modeling, uncertainty-aware digital twins, and decision-support systems for improving monitoring, prediction, optimization, and scale-up. Rather than presenting digital twins as autonomous solutions, it demonstrates that their value lies in integrating biological knowledge, heterogeneous data, mechanistic understanding, and uncertainty estimation within human-in-the-loop decision-support frameworks. The available evidence indicates that biological constraints, limited process observability, and insufficient integration of validated data remain the principal barriers to industrial implementation. In contrast, biology-aware, hybrid, and uncertainty-aware digital twins represent the most realistic direction for near-term deployment. Finally, techno-economic and life-cycle implications are discussed to highlight that industrial viability will depend not on a single technological breakthrough, but on the convergence of robust strains, resource-efficient cultivation, circular biorefineries, validated data infrastructures, and biology-informed AI tools. A roadmap for 2026–2036 is proposed to guide the transition of microalgal biofuels from laboratory promise toward industrial relevance. The integrated perspective presented here can guide future research and support the sustainable industrial deployment of microalgal biofuel systems.

## Graphical Abstract

**Figure:**
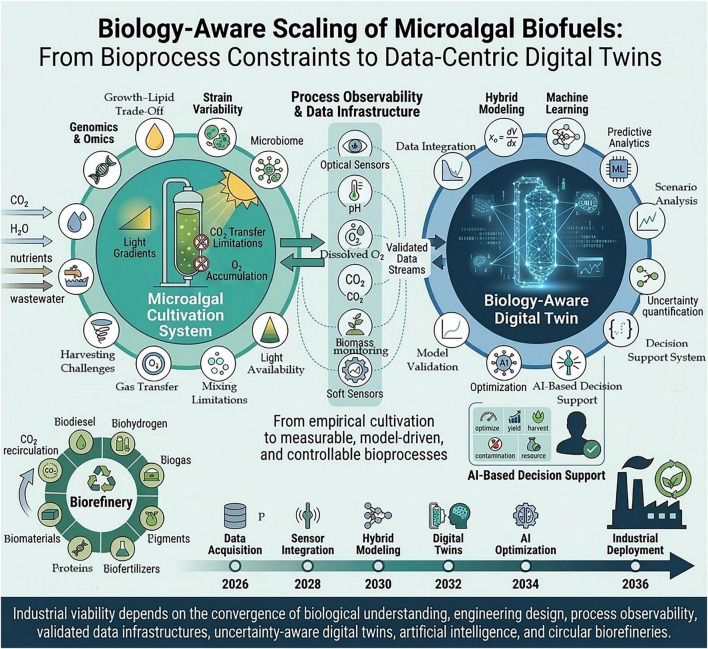
From bioprocess constraints in microalgal cultivation to biology-aware digital twins, integrating process observability, validated data streams, hybrid modeling, and artificial intelligence to enable predictive optimization, AI-assisted decision-making, and scalable circular microalgal biorefineries.

## Highlights

- Industrial-scale microalgal biofuels require biology-aware process observability rather than engineering optimization alone.
- Soft sensors and hybrid models enable inference of latent biological states for uncertainty-aware scale-up.
- A biology-aware roadmap integrates sensing, AI, digital twins, and techno-economic assessment for industrial microalgal biofuels.

## 1 Introduction

### 1.1 Challenges in scaling microalgal biofuels to industrial applications

The world energy system is changing rapidly, driven by the need to reduce GHG emissions, mitigate climate change, and ensure long-term energy security of supply (IPCC, 2023; Forster et al., 2024). Despite the continuous expansion of renewable energy, fossil fuels still dominate global energy consumption and have long been the primary source of CO_2_ emissions and environmental damage (Ezhumalai et al., 2024).

Conventional biofuels, including first- and second-generation biofuels derived from food crops and lignocellulosic biomass, face structural limitations arising from competition for land and water, as well as the complexity of conversion processes. These constraints have driven the development of third-generation biofuels, particularly those derived from microalgae, which represent a promising alternative. Microalgae efficiently utilize solar energy, CO_2_, and nutrients, and can achieve higher photosynthetic productivity than terrestrial crops (Chisti, 2007; Abdelfattah et al., 2023). They can be grown on non-arable land using brine or wastewater, with direct competition for food or fuel production, enabling integration with carbon capture and wastewater treatment (Novoveská et al., 2023).

Despite these advantages, large-scale industrial deployment remains limited, as light distribution, mass transfer, mixing, CO_2_ supply, and oxygen removal become increasingly difficult to control (Xu et al., 2024). Spatial and temporal gradients create microenvironmental heterogeneity that reduces the system’s productivity.

Scaling up microalgal cultivation involves complex interactions among physiology, metabolism, genetics, transport phenomena, environmental variability, and process control. Consequently, improving industrial performance requires an integrated biological and engineering approach rather than optimizing individual variables (Razzak et al., 2024).

Data-driven methods, such as hybrid modeling, digital twins, and machine learning (ML)-enabled automation, offer practical tools for managing microalgal systems. However, their application at industrial scale is still limited by data quality, model validation, and system integration (Porras Reyes et al., 2024; Sun et al., 2025). Their success ultimately depends on high-quality biological data, reliable instrumentation, and a mechanistic understanding of microalgal cultivation.

This review examines the biological, engineering, process observability, data, and economic constraints that currently limit the industrial deployment of microalgal biofuel systems. Particular attention is given to the transition from empirical optimization to data-centric, biology-aware process management enabled by advanced sensing technologies, artificial intelligence, hybrid modeling, and digital twins. These topics develop a systems-level perspective that identifies technological bottlenecks, knowledge gaps, and future opportunities for sustainable industrial scale-up.

### 1.2 Review scope and literature search

A systematic literature search was conducted in Scopus and the Web of Science Core Collection, supplemented by citation tracking. The search focused on publications from 2015 to 2026, with particular emphasis on advances reported between 2020 and 2026. The literature was selected based on its relevance to biological limitations, process monitoring and observability, sensing technologies, artificial intelligence, hybrid modeling, digital twins, and the techno-economic and sustainability aspects of industrial microalgal biofuel systems.

Included studies encompassed experimental, pilot-scale, and industrial investigations. Instead of providing an exhaustive overview, this work adopts a thematic and integrative perspective focused on scale-up constraints, knowledge gaps, and the development of biology-aware digital twins. This synthesis highlights the relationships among biological processes, engineering challenges, data collection, model development, and decision-support tools across scales.

## 2 Microalgae as bioenergy platforms

Microalgae serve as bioenergy platforms through three interconnected dimensions: cultivation configuration, metabolic strategy, and biomass valorization pathway. The performance and economic viability of microalgal systems depend not only on biomass productivity but also on the alignment between cultivation technology, conversion routes, and downstream valorization strategies (Brennan and Owende, 2010; Novoveská et al., 2023). Therefore, microalgal bioenergy systems should be analyzed as integrated platforms rather than as isolated production processes.

### 2.1 Cultivation configurations

Microalgal cultivation systems are primarily classified as open (open ponds, raceway ponds, and clarifiers) and closed (PBRs). Raceway ponds (RWPs) are widely used for large-scale biomass production due to their lower construction and operating costs compared to PBRs. Open systems harness sunlight through relatively simple mixing and circulation systems to reduce energy requirements (Carvalho et al., 2006). This makes them suitable for low-margin applications, wastewater treatment, and energy conversion of wet or residual biomass. However, their productivity is limited by environmental variability, biological contamination, low volumetric productivity, and limited control of temperature, pH, CO_2_ input, and nutrients (Skifa et al., 2025).

Closed photobioreactors (PBRs) provide greater control over cultivation conditions, such as light intensity and photoperiod, nutrients, temperature, pH, aeration, and CO_2_ supply, and reduce contamination risks (Razzak et al., 2024). Tubular, flat-plate, and bubbling-column PBRs can achieve higher cell densities and more consistent productivity than open systems. However, their widespread adoption is limited by high capital (CAPEX) and operating (OPEX) costs, energy consumption, overheating and biofouling, oxygen accumulation, shear stress, and difficulties with scaling and mass transfer (Hashmi et al., 2025). For this reason, PBRs are more suitable when combined with high-value products, carbon capture, or advanced control strategies.

Table 1 provides a comparative overview of the primary technical, economic, and operational characteristics of open RWPs, tubular photobioreactors, and flat-panel photobioreactors based on experimental studies, techno-economic analyses, industrial-scale reports, and review literature (Carvalho et al., 2006; Jorquera et al., 2010; Norsker et al., 2011; Novoveská et al., 2023; de Paula Pereira et al., 2024; Skifa et al., 2025).

**TABLE 1:** Comparative technical, economic, and operational characteristics of major microalgal cultivation systems for biofuel-oriented scale-up.

Parameter	Open raceway pond (ORP)	Tubular photobioreactor (PBR)	Flat-panel photobioreactor (PBR)
Biomass productivity (g m^−2^ d^−1^)	10–25	20–60	25–80
Volumetric productivity (g L^−1^d^−1^)	0.02–0.10	0.20–1.50	0.30–2.00
Typical biomass concentration (g L^−1^)	0.3–1.0	1–5	2–8
Biomass production cost including dewatering (€ kg^−1^ DW)	4.95	4.15	5.96
NER (biomass)	8.34	0.20	4.51
NER (oil)	3.05	0.07	1.65
CAPEX	Low	High	Very high
OPEX	Low–moderate	Moderate–high	High
Contamination risk	High	Low	Low
Environmental control	Limited	High	High
CO_2_ utilization potential	Moderate	High	High
Scalability	Commercially established ( > 100,000 m^2^)	Industrially demonstrated (35–100 m^3^)	Pilot-to-demonstration scale
Main engineering limitations	Contamination, evaporation, weather dependence, low culture density	Oxygen accumulation, biofouling, pumping energy demand, degassing requirements	Thermal management, wall fouling, cleaning requirements, scale-up complexity

CAPEX, capital expenditure; OPEX, operational expenditure; NER, net energy ratio.

As shown in Table 1, open raceway systems offer lower infrastructure costs and proven outdoor scalability than PBRs. On the other hand, tubular and flat-panel PBRs enable better environmental control and higher biomass concentrations, but with higher capital and operational costs. These trade-offs demonstrate the importance of a combined biological, engineering, and data-science approach to advancing process performance across scales.

Hybrid cultivation schemes, combining closed-system inoculum production with open-pond biomass expansion, offer a practical compromise between biological control and economic feasibility (Skifa et al., 2025). Pilot-scale studies have reported improved biomass productivity, mass transfer, contamination control, and operational flexibility through combined PBR-open pond systems (Liu et al., 2019; Dey et al., 2025; Kubar et al., 2025). Although hybrid systems have yielded encouraging results at the pilot and demonstration scales, large-scale validation beyond ∼1 ha remains limited, and most studies have been conducted on systems with capacities ranging from a few hundred liters to a few hundred square meters (Novoveská et al., 2023). Therefore, more hectare-scale demonstrations are needed to assess long-term operational stability, scalability, and techno-economic performance under industrially relevant conditions.

Because cultivation configuration directly affects environmental conditions, resource availability, and process economics, it also influences cellular metabolism, biomass composition, and the suitability of downstream conversion pathways.

### 2.2 Metabolic and conversion pathways

Metabolic strategy strongly influences biomass productivity. While autotrophic cultivation relies on light and CO_2_, mixotrophic cultivation combines photosynthesis with organic carbon assimilation, often increasing biomass productivity and facilitating the use of nutrient-rich waste streams (de Paula Pereira et al., 2024).

From a downstream processing perspective, microalgal biomass can be converted into different biofuels through biochemical, thermochemical, and catalytic pathways. Biodiesel remains the best-studied microalgal biofuel because many species accumulate fatty acids that can be made into alkyl esters via transesterification (Pandey et al., 2024). However, lipid accumulation is promoted under stress conditions that reduce biomass productivity, while harvesting, drying, extraction, and conversion contribute significantly to process costs (Chisti, 2007; Pandey et al., 2024). Hydrothermal liquefaction (HTL) has emerged as an attractive thermochemical route because it can process wet biomass without energy-intensive drying. Unlike biodiesel production, HTL utilizes the entire biomass fraction, making it suitable for heterogeneous feedstocks and wastewater-derived biomass (Hasan et al., 2024). However, the resulting biocrude requires upgrading before it can be used as fuel. Together, these pathways form complementary components of an integrated biomass valorization strategy rather than competing alternatives (Silva et al., 2024). The diversity of conversion pathways generates distinct requirements for monitoring, control, and optimization, which subsequently influence the design of sensing architectures, predictive models, and digital-twin frameworks.

### 2.3 Biorefinery integration and circular bioeconomy

The economic viability of biofuels derived from microalgal biomass depends on the biorefinery concept. Fuel production alone rarely offsets the costs of cultivation and downstream processing. Accordingly, microalgal biorefineries aim to integrate multiple utilization pathways to fractionate biomass into value streams, including biofuels, proteins, pigments, polyunsaturated fatty acids, biofertilizers, biochar, biomaterials, and bioactive compounds (Cheirsilp et al., 2023; Ezhumalai et al., 2024). The simultaneous or sequential recovery of these products improves overall profitability, diversifies revenue streams, and reduces waste.

Biorefinery strategies typically prioritize the recovery of high-value products before directing residual biomass toward energy applications such as anaerobic digestion, HTL, and biohydrogen production. This sequential valorization approach improves resource efficiency, the techno-economic feasibility of microalgal production systems, and supports circular economy models and closed-loop process architectures (Geng et al., 2025).

The industrial viability of microalgal biorefineries depends on the effective integration of cultivation, biomass conversion, by-product utilization, and resource recovery. Achieving industrial deployment, therefore, requires integrated cultivation, conversion, monitoring, and decision-support strategies (Chew et al., 2017; Ezhumalai et al., 2024).

## 3 Biological constraints governing microalgal biofuel production

Microalgal biofuels represent a sustainable alternative to fossil fuels. The unique biology of microalgae enables efficient CO_2_ fixation, high photosynthetic efficiency, and the accumulation of lipids as triacylglycerols (TAGs), which can subsequently be converted into biofuels (Brindhadevi et al., 2021; Bharti et al., 2022). However, despite their potential as third-generation biofuels, the commercial viability of the process remains limited by various aspects of microalgal biology. Lipid and biomass production are regulated by interconnected metabolic pathways involved in carbon allocation, oxidative stress, and cellular regulation (Khozin-Goldberg, 2016).

### 3.1 Physiological trade-offs between growth and lipid accumulation

One of the main biological challenges is the inverse relationship between cellular growth and lipid accumulation (Alishah Aratboni et al., 2019). Under optimal growth conditions, microalgae can produce between 20 and 50% lipids (dry weight). However, under stress conditions, lipid content can increase up to 70% (Morais et al., 2021; Suparmaniam et al., 2023). Nutrient depletion or increased light intensity reduces growth and biomass production while redirecting carbon fixation toward triacylglycerol (TAG) biosynthesis (Khozin-Goldberg, 2016; Wei et al., 2025).

Nitrogen (N) and phosphorus (P) deficiency are the nutrient stresses most used to stimulate lipid accumulation in microalgae. However, both stresses reduce biomass productivity (Maltsev et al., 2023). One approach is the simultaneous application of phytohormones and N-limitation. For example, Correa-Aguado et al. (2021) used benzylaminopurine (BAP) and gibberellic acid (GA) under N-limited conditions, resulting in fatty acid accumulation and increased biomass in *Scenedesmus obliquus*. The addition of 10^–5^ M BAP increased biomass 1.44-fold and total lipids 2.8-fold, while 10^–6^ M GA increased biomass by 1.35-fold and lipids by 1.11-fold, with maximum lipid yields of 55 and 50% (dry weight), respectively. Tamayo-Castañeda et al. (2025) combined phytohormone supplementation with N-limitation to increase carbon flux toward fatty acids and their conversion into TAGs in *Nannochloropsis oculata*. Under the optimized Box-Behnken conditions, biomass increased while lipid yield rose from 12.4 to 38.87%.

Another approach to overcoming the lipid-versus-biomass trade-off in microalgae is two-stage cultivation. This strategy separates biomass production from lipid induction by applying nutrient-replete growth followed by stress conditions. In *Chlorella* sp., this strategy achieved a lipid content of 36.7% and a productivity of 216.9 mg L^–1^ d^–1^ after 5 days of N-replete growth followed by 4 days of N-limitation (Nayak et al., 2019; Aziz et al., 2020).

The two-step approach can improve lipid productivity. However, the N-reset phase increases energy and operating costs. Nutrient manipulation, biomass transfer, and additional monitoring may increase water and operating costs, depending on the cultivation system (Leal et al., 2026; Xu et al., 2026).

Although two-stage cultivation can increase both biomass and lipid production, optimal conditions remain species-dependent. Therefore, the growth-lipid trade-off remains one of the main biological bottlenecks limiting the transition from laboratory productivity to industrial-scale production.

### 3.2 Metabolic plasticity and stress responses

Metabolic plasticity enables microalgae to adapt to changing environmental conditions by adjusting their metabolic pathways (Khozin-Goldberg, 2016). This flexibility arises because lipid biosynthesis depends on how photosynthetically fixed carbon is allocated among competing metabolic pathways. Microalgae can integrate light and CO_2_ signals to regulate processes such as photosynthesis, carbon fixation, and energy storage (Yamaoka et al., 2025). Carbon allocation is therefore determinant of lipid biosynthesis because cells prioritize growth and maintenance under favorable conditions (Li et al., 2025). Although metabolic plasticity contributes to environmental adaptation, it also introduces nonlinear responses that complicate the prediction, monitoring, and control of processes in large-scale cultivation.

### 3.3 Interspecies and intraspecies variability

Microalgae exhibit substantial physiological and metabolic variability among genera, species, and even strains within a species. This variability is reflected in differences in the production of lipids, pigments, and other metabolites. Natural differences in their genomes result in diverse photosynthetic responses and stress tolerances. Most wild-type strains do not simultaneously combine high lipid content, rapid growth, and the characteristics required for industrial production (Metting, 1996). However, identifying strains with optimal traits for specific biotechnological applications remains a major challenge. Addressing this challenge requires selecting wild strains with desirable characteristics and applying genetic engineering tools to enhance specific traits (Chen et al., 2020; Craig et al., 2021).

Most studies addressing these particularities across strains and species are conducted primarily in the laboratory, where microalgal cultures can be grown under optimal conditions. However, when these studies are scaled up to pilot or commercial levels, natural factors can significantly alter results obtained under laboratory conditions. These factors include variable light conditions, interactions with other microorganisms, temperature and pH fluctuations, and nutrient availability (Alishah Aratboni et al., 2019). Temperature is a critical parameter for controlling microalgal growth. Most species grow best between 20 and 30°C; thermal requirements vary substantially among taxa and even among strains of the same species (Dȩbowski et al., 2022). A recent compilation of cardinal temperatures for 424 microalgal, cyanobacterial, and diatom strains demonstrated considerable variability in optimal growth temperatures across taxa. Consequently, deviations from strain-specific temperature optima can severely affect photosynthesis, metabolism, and biomass productivity (Rossi et al., 2023).

### 3.4 Microalgae-microbiome interactions in non-sterile systems

Microalgae-microbiome interactions are relevant during scale-up because industrial systems are rarely sterile. Microalgae coexist with diverse microbial communities that may either support or hinder cultivation performance through nutrient competition, metabolic interactions, or pathogenic effects (Dȩbowski et al., 2020). As a result, microbial community dynamics become a source of uncertainty during scale-up and represent an important challenge for process stability, monitoring, and predictive modeling.

Taken together, these biological constraints show that microalgal cultivation is not only an engineering challenge but also a biological one. Physiological trade-offs, metabolic plasticity, strain variability, and microbiome interactions strongly influence productivity and become increasingly important during scale-up. Their successful integration into engineering design, process monitoring, and predictive models will be essential for reliable industrial cultivation.

## 4 Engineering and operational constraints at large scale

**FIGURE 1:**
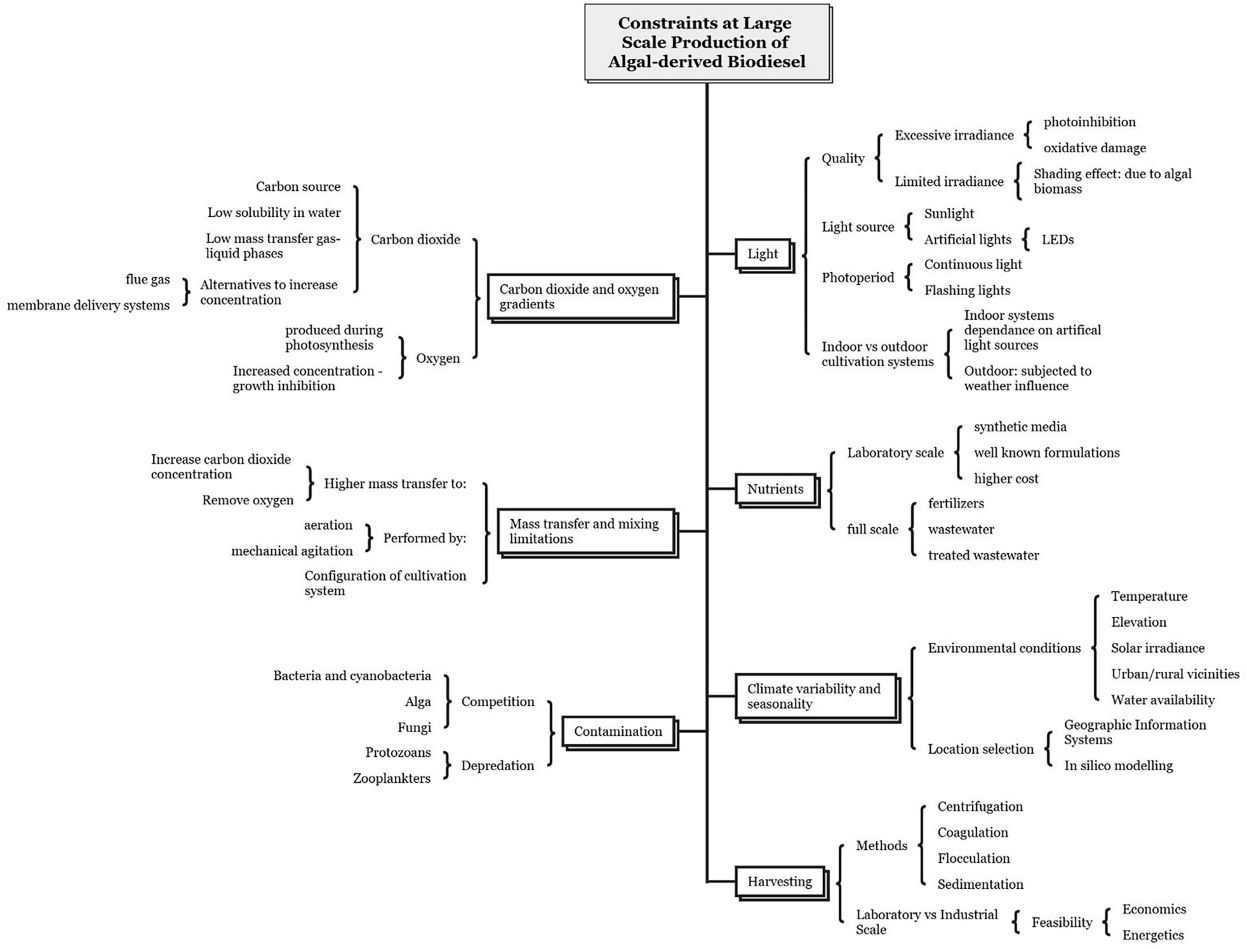
Overview of the main biological, environmental, and engineering constraints affecting large-scale microalgal biodiesel production. These interconnected factors influence biomass productivity, process stability, scalability, and economic feasibility.

Scaling up microalgal cultivation is essential for industrial profitability. In laboratory-scale systems, all environmental variables are controlled to maximize biomass and lipid production. However, industrial-scale cultivation introduces engineering challenges related to light attenuation, CO_2_ depletion, O_2_ accumulation, pH fluctuations, mixing energy requirements, and biomass harvesting, all of which reduce process efficiency and profitability (Razzak et al., 2024). Therefore, scaling up from laboratory systems to open ponds, RWPs, and PBRs requires addressing these engineering constraints (Grobbelaar, 2012; Avinash et al., 2020). Figure 1 summarizes the main biological, environmental, and engineering constraints discussed throughout this section.

### 4.1 Light gradients

In large-scale systems, light availability becomes heterogeneous because dense cultures generate self-shading and strong spatial gradients. While PBRs allow greater control of irradiance and photoperiod, outdoor ponds depend on sunlight, seasonality, and geographic location. Reactor design and mixing strategies must minimize light gradients while maintaining efficient photosynthetic conditions and energy efficiency (Béchet et al., 2016; Legrand et al., 2021; Vasile et al., 2021).

Several studies have shown that improving light distribution substantially increases biomass productivity. For example, in flat-plate gas-lift photobioreactors, optimizing light distribution increased areal productivity by 113% to 10.4 g m^–2^ d^–1^ in *Nannochloropsis salina* and by 107% to 14.8 g m^–2^ d^–1^ in *Nannochloropsis gaditana* (Pfaffinger et al., 2016). In addition, strategies developed to improve light distribution increased photosynthetic efficiency from 4.8 to 5.6% and the specific growth rate from 0.30 to 0.35 d^−1^ (Tredici and Zittelli, 1998). Carlozzi (2003) developed an outdoor tubular undulating-row photobioreactor for *Arthrospira* with a surface-to-volume ratio of 400 m^–2^, achieving an aerial productivity of 47.7 g m^–2^ d^–1^. These results show that productivity depends not only on incident irradiance but also on optical path length, light attenuation, reactor geometry, and cell circulation.

### 4.2 Carbon dioxide and oxygen gradients

Carbon dioxide availability and oxygen accumulation are major limitations in large-scale microalgal cultivation. Although CO_2_ is the primary carbon source for autotrophic growth, its transfer from the gas phase to the culture medium is often inefficient because of its low solubility and short residence time (Fu et al., 2019; Hajinajaf et al., 2024). As a result, CO_2_ supplementation through air enrichment, flue gas utilization, or bicarbonate addition is commonly required to sustain biomass productivity and carbon fixation rates (de Godos et al., 2014; Razzak et al., 2024). Simultaneously, oxygen generated during photosynthesis can accumulate within the culture and reach inhibitory levels at high dissolved concentrations. Excess dissolved oxygen reduces photosynthetic efficiency and may negatively affect biomass productivity, particularly in dense cultures and poorly mixed systems. Adequate aeration, mixing, and hydrodynamic design are required to balance CO_2_ supply, O_2_ removal, and energy consumption (Masojídek et al., 2021; Skifa et al., 2025).

Gas-liquid (G-L) transfer remains a major bottleneck in large-scale growth systems. Recent experimental and CFD-validated studies reported volumetric mass-transfer coefficients (kLa) ranging from 0.003 to 0.008 s^–1^ in airlift PBRs and up to 0.037 s^–1^ in optimized open-tube airlift configurations (Rezvani and Rostami, 2023; Hajinajaf et al., 2024; Emamshoushtari et al., 2026). These results highlight the influence of reactor geometry, gas holdup, bubble size, and hydrodynamics on CO_2_ transfer efficiency.

In high-rate algal ponds, atmospheric exchange supplies only about 5% of the carbon required for photosynthetic growth, whereas conventional CO_2_ sparging may lose 80–90% of the injected CO_2_. Carbonation columns have achieved a transfer efficiency of approximately 83% by extending G-L contact time (Putt et al., 2011). Increasing carbon supply alone is not enough; high O_2_/CO_2_ ratios induce photorespiration and reduce the efficiency of carbon fixation (Masojídek et al., 2021). Photobioreactor productivity depends on effective CO_2_ supply, O_2_ removal, and gas-transfer management (Fu et al., 2019; Masojídek et al., 2021).

### 4.3 Nutrients

Laboratory media are optimized for the growth of specific microalgal species, maximizing biomass and lipid production. However, these formulations are economically impractical at an industrial scale, necessitating alternative nutrient sources rather than analytical-grade reagents. Domestic and treated wastewaters can serve as cultivation media because they contain CO_2_, N, and P while simultaneously mitigating nutrient discharge and eutrophication (Masojídek et al., 2021). Coupling wastewater treatment with microalgal cultivation reduces biomass production costs, nutrient discharge, and freshwater demand while improving biomass productivity. However, large-scale application still requires monitoring of nutrient variability, pathogens, and competing microorganisms, as well as regular cleaning of cultivation systems (Bradley et al., 2023).

Large-scale production must account for nutrient variability and availability, as well as process reproducibility. Reported removal efficiencies range from for microalgae systems treating domestic, industrial, and agricultural wastewaters: Chemical Oxygen Demand (COD) removal, from 38.5 to 82%; Total Nitrogen (TN) removal, from 67.11 to 99.0%; and Total Phosphorus (TP) removal, from 79 to 99.1%, with biomass concentrations of 0.05–5.1 g/L (Kong et al., 2025). Municipal wastewater systems can also achieve high N and P removal efficiencies of 72.0–96.9 and 88.2–99.8%, respectively. However, pilot-scale results are inconsistent; for example, high ammonium-nitrogen removal (99.86%) and much lower mean TP removal (25.69%) (Popa et al., 2025). These findings indicate that the main engineering challenge is sustaining biomass productivity while recovering nutrients across variable wastewater matrices and operating conditions.

### 4.4 Mass transfer and mixing limitations

Efficient mixing is essential in large-scale cultivation because it enhances CO_2_ transfer, removes excess oxygen, reduces sedimentation, and mitigates self-shading. However, mixing is energy intensive. Excessive agitation may generate shear stress and negatively affect physiology, whereas insufficient mixing promotes biomass aggregation and heterogeneous conditions that reduce productivity (He et al., 2022).

Reactor geometry strongly influences this trade-off. PBRs generally provide greater process control but often require higher energy inputs to maintain adequate circulation and gas transfer. In contrast, RWPs achieve mixing through paddle wheels and offer lower operating costs, although hydrodynamic limitations and dead zones may reduce performance. Optimizing mixing and mass transfer remains essential for improving productivity and economic viability during scale-up (Hoeniges et al., 2022; Skifa et al., 2025; Silkina and Gayo Pelaez, 2026).

Chiaramonti et al. (2013) demonstrated that replacing a paddle wheel with a propeller in RWPs reduced power demand from 82.5 to 28.8 W, resulting in a 65% energy saving, and achieved high microalgal productivity. Scale-up estimates indicated a specific consumption of ∼0.47 W m^−2^ and a 60.5% energy saving compared to a conventional raceway pond. Mechanical agitation can increase mass-transfer coefficients by 30–60%, but the energy requirements may range from 0.1 to 3.0 kWh kg^−1^ of biomass, and transfer efficiency can decrease by 30–60% during scale-up without design modifications. One of the major challenges is achieving adequate CO_2_ transfer, O_2_ removal, and cell circulation with minimal energy input, while avoiding cell damage from excessive shear stress and/or reductions in cell viability (Wang and Lan, 2018; Obidi and Bayless, 2025).

### 4.5 Environmental and biological disturbances

Temperature affects algal growth in several ways (Skifa et al., 2025). Low temperatures slow enzymatic reactions, reducing growth and cell division. During early daylight hours, photosynthetic oxygen production may exceed its consumption, increasing the risk of photoinhibition. Biomass productivity varies seasonally according to temperature and day length.

Seasonal differences become evident when productivity is measured across year-round cultivation cycles. Under temperature-controlled outdoor simulations, areal biomass productivities ranged from 7.9 g m^–2^ d^–1^ in winter to 31.8 g m^–2^ d^–1^ in summer, representing a four-fold seasonal difference. Moreover, nutrient limitation resulted in a 7–16% reduction in winter productivity and up to 60% in summer productivity, highlighting the interaction between the environment and culture physiology (Huesemann et al., 2023). Recent forecasting methods achieved 74–84% prediction accuracy and biomass gains exceeding 15%, supporting climate-adaptive cultivation strategies for industrial cultivation (Yan et al., 2026).

Closed cultivation systems inoculated with defined algal strains are less susceptible to contamination than open systems such as open ponds and open raceways. Biological contamination includes competitors for nutrients and light, grazers, allelopathic organisms, and parasitic fungi or viruses. Contamination events can significantly reduce biomass productivity and product quality, highlighting the need for robust monitoring and early detection strategies in large-scale cultivation systems (Lam et al., 2018).

### 4.6 Harvesting

Harvesting separates algal biomass from the culture medium and remains challenging because cultivation systems typically contain only 0.5–5 g/L biomass (Molina Grima et al., 2003; Uduman et al., 2010). Available methods include centrifugation, gravity sedimentation, and flocculation, but not all are applicable at an industrial scale due to their high costs. Although centrifugation provides the highest recovery efficiency, its cost limits industrial applications. Therefore, gravity sedimentation, coagulation, and flocculation are preferred for their lower energy demand (Sharma et al., 2025). After concentration, drying strategies should be selected based on energy demand, biomass characteristics, and cultivation conditions, including axenic or co-culture systems (Gaurav et al., 2024). Recent techno-economic assessments illustrate this trade-off: filter harvesting achieved higher concentration factors than flocculation-based methods (770–1,086 vs. 407–448) but also demanded significantly greater energy input (4.343 vs. 0.389 kWh m^–3^) and slightly greater costs (5.35 vs. 4.52 USD m^–3^) (Lu et al., 2022). Harvesting optimization should consider recovery efficiency, biomass quality, energy consumption, and operating costs, rather than simply maximizing biomass concentration.

## 5 Genetic and omics-based foundations for predictive microalgal bioprocesses

### 5.1 Genetic basis of lipid accumulation in microalgae

Genetic differences among microalgal species and strains largely determine lipid accumulation capacity. For example, *Scenedesmus obliquus* strains with biodiesel production potential are efficient at producing TAGs due to their lipogenic genes and epigenetic regulation (Chen et al., 2020). Specifically, they have been shown to overexpress lipid synthesis genes, such as Acyl Carrier Protein (ACP), Stearoyl-ACP Desaturase (SAD), Fatty Acyl-ACP Thioesterase A (FATA), and Diacylglycerol Acyltransferase (DGAT), resulting in up to a 55% increase in the production of the biodiesel-relevant fatty acids C16:0 and C18:1. This was achieved by subjecting the culture to nitrogen-limited stress, while simultaneously supplementing the medium with growth regulators to conserve growth and biomass (Correa-Aguado et al., 2021). However, lipid accumulation is not controlled by individual genes alone but by coordinated metabolic networks that require NADPH and ATP. This coordination involves processes such as the Calvin cycle, respiratory metabolism, the pentose phosphate pathway, and lipid biosynthetic pathways. Genes encoding enzymes involved in reducing power generation, energy production, and the supply of metabolic precursors for fatty acid synthesis participate in these processes (Yu et al., 2011; Wang et al., 2024; Yamaoka et al., 2025). Recent genome-scale and constraint-based metabolic models also support this view and indicate that lipid accumulation is the result of global flux redistribution and carbon partitioning over photosynthesis, central carbon metabolism, nitrogen assimilation, acetyl-CoA supply, NADPH production, and triacylglycerol (TAG) biosynthesis rather than a single lipogenic reaction (Li et al., 2019; Ray et al., 2023; Tamburro and Boyle, 2025). Lipid productivity is regulated by multiple pathways, necessitating systems-level approaches that capture biological complexity in predictive cultivation models.

### 5.2 Metabolic regulation and key biosynthetic pathways

Lipid accumulation in microalgae results from a complex network of metabolic regulation encompassing several biosynthetic pathways that adapt to environmental and physiological factors (Khozin-Goldberg, 2016; Kong et al., 2024). This regulation depends on the presence of lipogenic enzymes, the availability of precursors, carbon fixation, and the genetic regulation of these pathways. Under optimal growth conditions, the carbon fixed during photosynthesis is used to synthesize proteins and carbohydrates, thereby supporting cell growth. However, under stress conditions, metabolic reprogramming occurs, directing carbon toward lipid biosynthesis, primarily triacylglycerols (TAGs) (Khozin-Goldberg, 2016; Bharti et al., 2022; Yamaoka et al., 2025). Several key regulatory steps have been identified in fatty acid biosynthesis, involving enzymes such as acetyl-CoA carboxylase (ACCase), which participates in the initial step of fatty acid biosynthesis; acyl-ACP thioesterases, which are involved in determining fatty acid chain length; and diacylglycerol acyltransferases (DGATs), which participate in the final step of TAG synthesis (Yu et al., 2011). These enzymes represent promising targets for genetic engineering to improve lipid productivity. However, the regulation of these enzymes is complex, and blocking one pathway can activate undesirable compensatory pathways. Effective strain improvement strategies require systems-level understanding of metabolic interactions rather than the manipulation of single targets. This complexity also supports the use of computational models that integrate metabolic regulation into predictive frameworks.

### 5.3 Contributions from bioinformatics, genomics, transcriptomics, and proteomics

In recent years, the integration of omics science and systems biology has led to a deeper understanding of the genetic foundations of lipid metabolism. The sequencing of genomes, including those of *Chlamydomonas* (Craig et al., 2021), *Scenedesmus obliquus* (Chen et al., 2020), and *Haematococcus pluvialis* (Luo et al., 2019), has revealed similarities and differences in gene organization among species. When combined with bioinformatics, these advances enable prediction of genetic modifications using computational algorithms and three-dimensional modeling before laboratory implementation, helping optimize genetic engineering strategies. Thus, metabolic engineering, supported by omics sciences and bioinformatics, enables a safer, more rational approach by targeting the overexpression or silencing of regulatory genes and proteins involved in the biosynthesis of fatty acids and triacylglycerols for biofuel production (Wei et al., 2025).

Beyond strain engineering, omics datasets are increasingly used as data layers for data-driven and hybrid modeling approaches. Transcriptomic, proteomic, and metabolomic information can help identify biological state variables, improve mechanistic model calibration, and support the development of biology-aware digital twins that represent cellular responses under changing cultivation conditions.

## 6 Experimental observability

Industrial microalgae cultivation combines biotechnology, fluid dynamics, and mechatronics engineering. As interest in the sustainable production of biomass for biofuels, nutraceuticals, and carbon sequestration grows, the need for precise process control has increased. Microalgae are photosynthetic organisms capable of transforming light, carbon dioxide, and inorganic nutrients into complex organic compounds. However, the biological variability of these microorganisms, coupled with the physical limitations present in large-scale PBRs, requires a better understanding of relevant process variables, as well as monitoring technologies and automated control systems (Ryalat et al., 2024; Sangregorio-Soto et al., 2026).

### 6.1 Critical process variables

**FIGURE 2:**
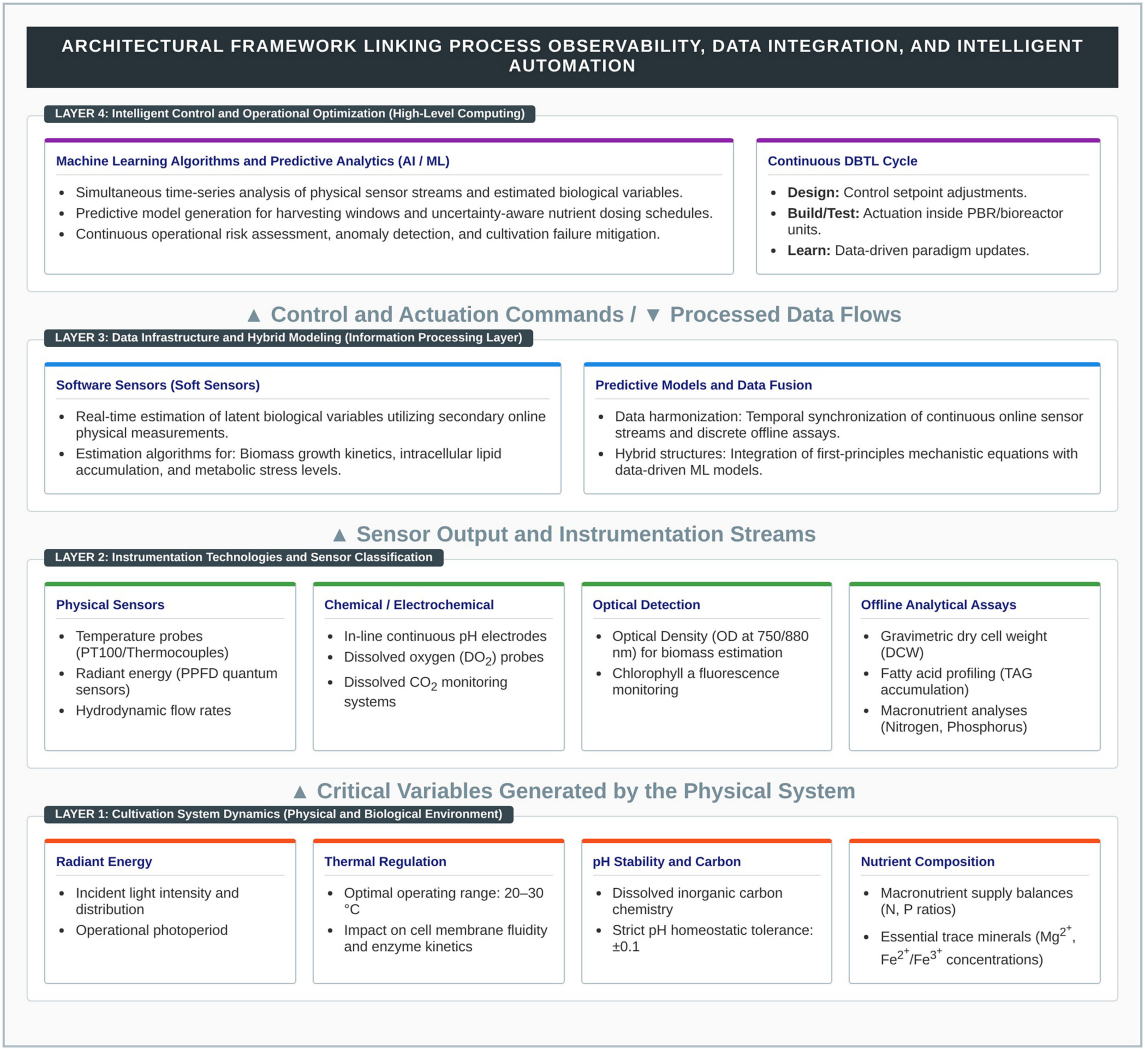
Conceptual architecture linking process observability, sensing technologies, data integration, and intelligent automation for biology-aware microalgal cultivation systems. The framework illustrates the hierarchical flow from critical process variables to predictive monitoring, decision support, and Digital Twins.

Optimizing microalgal growth requires balancing interconnected environmental and chemical parameters. For example, carbon dioxide uptake depends on both light intensity and temperature. When these conditions fall outside their optimal ranges, problems such as culture collapse, reduced lipid production, or the emergence of biological contaminants may occur (Chanquia et al., 2022; Gao et al., 2024). The monitoring and control of microalgal cultivation systems rely on the integration of critical process variables, sensing technologies, data acquisition, and automation strategies. All these components underpin modern observability frameworks that support process optimization, predictive analytics, and intelligent cultivation management. Figure 2 illustrates how these components interact to support process observability and intelligent cultivation management.

#### 6.1.1 Radiant energy

From an observability perspective, radiant energy is commonly monitored through photosynthetic photon flux density (PPFD), whose relationship with growth rate is nonlinear and follows a characteristic saturation curve. When light intensity is low, the system operates below the compensation point, where respiration exceeds photosynthesis and, as a result, biomass loss occurs (Sharma et al., 2025). As light intensity increases, growth rises nearly linearly until saturation is reached. Beyond this limit, microalgae may experience photoinhibition, which affects productivity and should therefore be considered in monitoring and control strategies (Huang et al., 2026).

#### 6.1.2 Thermal regulation

Temperature is a decisive factor in microalgal development, as it directly influences enzymatic activity and cell membrane fluidity. Many commercially important species, such as *Chlorella* and *Arthrospira*, exhibit optimal growth within a temperature range of approximately 25–30°C (Chanquia et al., 2022). Temperatures outside this range can severely affect culture performance. High temperatures increase cellular respiration and can cause protein denaturation, while low temperatures reduce metabolic activity and slow growth to the point of making it uneconomical (Ouyang et al., 2023).

#### 6.1.3 Carbon assimilation and pH stability

Carbon accounts for about 50% of the dry weight of microalgal biomass. In autotrophic systems, carbon is generally supplied by the injection of carbon dioxide. The concentration of dissolved carbon is closely related to the pH of the culture medium because dissolved CO_2_ forms carbonic acid, which subsequently dissociates into bicarbonate and carbonate ions, lowering pH (Dȩbowski et al., 2025). Precise control of pH, generally within a tolerance of ± 0.1, is essential not only to maintain adequate carbon availability but also to ensure that nutrients remain in ionic forms and remain available for uptake by microalgae (Fuchs et al., 2021; Chanquia et al., 2022).

#### 6.1.4 Composition of the medium

The nutritional requirements of microalgae are closely related to their cellular composition, which is often represented by an empirical molecular formula. Nitrogen (N) and phosphorus (P) are the most important macronutrients for microalgal growth (Ragueneau et al., 2026). High N concentrations typically promote rapid biomass production; however, N-limitation is also widely used as a stress strategy to stimulate the synthesis of reserve lipids or high-value compounds, such as carotenoids and astaxanthin (Dȩbowski et al., 2025).

### 6.2 Classification of instrumentation technologies

The ability to monitor critical culture variables in real time is fundamental to modern bioprocess control. In microalgae cultivation, measurement technologies are typically classified into physical, chemical, and biological sensors. They can also be differentiated based on their mode of operation, whether online, continuous online, in-line, or offline (Havlik et al., 2022; Porras Reyes et al., 2024).

#### 6.2.1 Physical instrumentation

Physical sensors enable the measurement of basic environmental conditions within the bioreactor. Among the most commonly used are thermocouples and PT100-type temperature sensors, which are employed for thermal monitoring and are increasingly integrated into IoT systems for remote data acquisition (Havlik et al., 2022). Physical instrumentation includes temperature, flow, pressure, and light sensors that support environmental monitoring and process control in photobioreactors (Chanquia et al., 2022; Zakova et al., 2025).

#### 6.2.2 Chemical and electrochemical sensors

Chemical sensors are used to monitor the aqueous medium and nutrient availability. Among these, the pH electrode serves not only to measure acidity but also as an indirect indicator of biological activity and dissolved carbon concentration. Likewise, dissolved oxygen and carbon dioxide sensors are essential for performing mass balances and evaluating photosynthetic activity (Jia et al., 2015; Porras Reyes et al., 2024).

#### 6.2.3 Optical detection

Biological sensors provide direct insights into the physiological status of microalgae. Optical density (OD) sensors are used for real-time biomass monitoring and estimation. These devices measure light attenuation, typically at wavelengths where pigment interference is minimal, such as 750 or 880 nm (Plouviez et al., 2026). By correlating optical density with dry cell weight, cultures can be maintained in specific growth phases using automated dilution strategies, such as in turbidostat-type systems (Cotta et al., 2025).

#### 6.2.4 Software sensors

Software sensors estimate biological variables that cannot be measured directly in real time by combining physical measurements with mathematical or data-driven models. These approaches have become an important component of precision phyco-culture because they provide continuous estimates of intracellular compounds, physiological status, productivity indicators, and other latent biological variables that are difficult to monitor experimentally. By reducing dependence on labor-intensive offline analyses, software sensors improve temporal resolution and support process monitoring, optimization, and automated control strategies (Havlik et al., 2022; Porras Reyes et al., 2024).

### 6.3 Stress detection and physiological status

Automated online estimation using software sensors enables continuous monitoring of the culture status, facilitating early detection of stress conditions in microorganisms and helping prevent damage or production losses.

Thanks to continuous data collection, these systems serve as early warning mechanisms, enabling adjustments to bioreactor parameters (such as lighting, temperature, or nutrient concentration) before stress significantly affects crop productivity (Porras Reyes et al., 2024; Xia et al., 2026).

### 6.4 Practical limitations and measurement challenges

The transition from controlled laboratory experiments to industrial microalgae cultivation systems presents various challenges in measurement and monitoring. These difficulties arise primarily from the interaction between the biological medium and the physical sensors used in the process (Zakova et al., 2025).

#### 6.4.1 Biofouling and signal degradation

Biofouling is a common problem in monitoring systems. Within a few hours of a sensor being submerged in the culture, macromolecules and pioneer bacteria begin to adhere, followed later by microalgal cells and filaments (Chen et al., 2025). The formation of this biofilm directly affects the quality of measurements. In optical sensors, for example, the accumulation of biomass on the reading windows can lead to erroneous estimates of high cell density. Similarly, in chemical sensors such as pH or dissolved oxygen sensors, the biofilm creates microenvironments around the sensor that alter the actual readings. As a result, fouled sensors may reflect conditions within the biofilm rather than those of the surrounding culture, leading to errors in automatic control, aeration, and dosing (Delgado et al., 2021).

The first 48 h are often fundamental for biofilm formation, and if mitigation strategies are not implemented, some sensors may lose their calibration in less than a week. Among the most commonly used solutions are mechanical cleaning systems, copper alloy coatings with a biocidal effect, and physical techniques such as ultrasonic irradiation to dislodge adhered cells (Chen et al., 2025).

#### 6.4.2 Light attenuation and signal noise in dense media

As the concentration of microalgae increases, the culture becomes more opaque, reducing light penetration. This behavior follows Beer-Lambert’s law, whereby light intensity decreases exponentially with depth and cell concentration. In high-density industrial cultures, effective light penetration can be reduced to just a few millimeters, resulting in very low signal-to-noise ratios for conventional optical sensors. To overcome this limitation, specialized sensors with short optical paths and high-intensity NIR (near-infrared) light sources are used, enabling linear, accurate measurements without the need to manually dilute the samples (Sazandehchi et al., 2025).

Sensor performance in large-scale PBRs can be affected by several physical and biological phenomena. The main limitations and mitigation strategies are summarized in Table 2.

**TABLE 2:** Factors affecting sensor performance in photobioreactors.

Sensor	Physical/biological mechanism	New mitigation strategies
Biofouling	Rapid cell adhesion and biofilm formation through the secretion of extracellular polymeric substances (EPS) (Hashmi et al., 2025).	1. Cell signaling interruption (Quorum Quenching) using immobilized bacteria (Hashmi et al., 2025). 2. Coatings of polyacrylamide hydrogels and zwitterionic polymers (Chan et al., 2022). 3. Biomimetic topographies (moth-eye nanoholes) and photocatalytic nanofilms. 4. Detachment triggered by mechanical stress or ultrasound (Hashmi et al., 2025).
Attenuation (Light scattering)	Severe light refraction and scattering at the liquid-bubble interface during dense aeration.	1. Automated bubbling interruption mediated by solenoid valves. 2. Hydrophobic cutting slices to fractionate gas into microbubbles imperceptible to the optics (Zhao et al., 2023).
Drift	Photochemical degradation of indicator dyes, anode oxidation, and internal electrolyte depletion in electrochemical probes.	1. Transition to solid-state optical transducers (fluorescence quenching optodes). 2. AIoT soft sensors with DTKNN+ hybrid frameworks for algorithmic computational drift compensation (Stine et al., 2020).
Spectral and chemical interference	Overlap of spectral signatures, accumulation of metabolic byproducts, and microflora complexity.	1. Digital twins and multivariate data fusion using ML algorithms such as support vector machines (SVM) and CNN-LSTM networks (Otálora et al., 2024).
Thermal and transport inertia	Slow heat/mass transfer across the boundary layer in large volumes and thick biofilms.	1. Advanced model predictive control (MPC), such as the 3DoF-KF MoD filter. 2. Long short-term memory (LSTM) neural networks to predict temporal dependencies and automatically adjust flows (Kabir et al., 2026).

## 7 From bioprocess knowledge to data requirements

The development of data-driven tools for microalgal biofuels must be grounded in biological and process knowledge to define what needs to be measured, inferred, documented, and validated. Microalgal cultivation is a bioprocess in which cell growth, lipid accumulation, photosynthetic efficiency, stress responses, biochemical composition, and culture stability result from interactions between environmental factors and cellular physiology (Masojídek et al., 2021).

Recent studies have shifted from evaluating the technical feasibility of microalgal biofuels to exploring how AI, ML, software sensors, intelligent monitoring, hybrid modeling, and computational workflows can support cultivation, harvesting, conversion, and scale-up (Yang et al., 2011; Coşgun et al., 2023; Imamoglu, 2024; Syed et al., 2024). These reviews indicate that AI-driven methodologies are no longer marginal in microalgal biofuel research. They also highlight a fundamental limitation: the bottleneck is not so much the lack of algorithms as the lack of standardized, representative, well-documented, reusable datasets. The central challenge has shifted from demonstrating technical feasibility to developing transferable, data-rich frameworks that support prediction, optimization, and scale-up across biological and operational contexts.

A large body of scientific literature does not automatically yield usable data. Many studies generate valuable information within a specific experimental setting. However, these data are not always reusable across strains, reactors, scales, media, or environmental conditions. A model trained under one experimental configuration may not generalize to another, even when the same variables are measured (Bernard, 2011; Yang et al., 2024). In microalgal systems, the interpretation of a variable depends strongly on strain identity, cultivation mode, reactor geometry, light regime, nutrient availability, operational conditions, sampling frequency, analytical method, and measurement protocol.

Biology-aware scale-up begins with biology-aware data requirements. In microalgal biofuel systems, data are meaningful only when they preserve the biological and process context from which they emerge. This includes directly measurable variables, indirectly inferred biological states, operational metadata, scale descriptors, and indicators of uncertainty and reproducibility.

### 7.1 Information generated by biological and process systems

Microalgal biofuel production systems generate information through the interaction between biological activity and process operation. The biological layer includes biomass accumulation, cell density, growth rate, lipid accumulation, pigment composition, biochemical profile, viability, morphology, photosynthetic efficiency, stress responses, and indicators of contamination (Masojídek et al., 2021). The process layer includes light availability, temperature, pH, dissolved oxygen, CO_2_ supply, mixing, nutrient concentration, salinity, hydraulic retention time, reactor configuration, and operating mode (Imamoglu, 2024; Porras Reyes et al., 2024).

A biology-aware data strategy should distinguish between variables that directly describe the culture, variables that describe the environment imposed on the culture, and variables that explain the interaction between the two layers. This distinction is essential because scale-up changes the relationship between nominal operating conditions and the effective cellular environment experienced by microalgae. For example, light intensity is more than an engineering input; it determines photosynthetic exposure, self-shading, photo-inhibition, and productivity potential. Similarly, pH is not only a chemical measurement; it reflects carbon chemistry, metabolic activity, and process stability (Barajas-Solano et al., 2026).

Table 3 summarizes the principal biological and process data layers required for biology-aware digital tools in microalgal biofuel systems. The classification integrates concepts from microalgal cultivation monitoring, process observability, hybrid modeling, and AI-assisted bioprocess analysis, and highlights the typical temporal resolutions of different data streams (Masojídek et al., 2021; Porras Reyes et al., 2024).

**TABLE 3:** Conceptual framework summarizing biology-aware data requirements for microalgal biofuel systems.

Data layer	Examples	Biological or process meaning	Implication for data requirements	Typical measurement frequency
Biological state	Biomass, cell density, growth rate, viability, morphology, photosynthetic efficiency, contamination indicators	Describes culture performance and physiological status	Requires biological measurements or validated proxies	Periodic measurements; high-frequency optical monitoring when available
Biofuel-related composition	Lipids, carbohydrates, proteins, pigments, intracellular products, biochemical profile	Determines energy potential, metabolic allocation, and harvest timing	Often requires offline or indirect measurements	Periodic laboratory analyses or indirect estimation
Physicochemical environment	pH, temperature, dissolved oxygen, dissolved CO*2*, nitrogen, phosphorus, salinity	Defines the immediate environment that shapes growth, stress, and productivity	Should be linked to biological outputs and sampling	Continuous or high-frequency sensor monitoring
Operational layer	Light intensity, mixing, reactor type, medium composition, CO_2_ flow	Biological trajectories	Requires complete operational metadata and consistent units	Continuous logging or event-based recording
Scale-dependent layer	Light-path length, self-shading, gradients, hydrodynamics, fouling	Explains why laboratory performance may not transfer to pilot or industrial scale	Requires spatial, temporal, and geometry-aware descriptors	Periodic assessment or model-based estimation
Latent biological states	Cellular stress, intracellular lipid dynamics, local light exposure, contamination risk	Vital states are often hidden, indirect, or only partially observable	Requires validated proxies, software sensors, and uncertainty descriptors	Model-based or software-sensor estimation

Measurement frequencies are indicative and depend on cultivation system design, sensor availability, process dynamics, and monitoring objectives.

This means that data requirements extend beyond sensor availability. They must preserve the connection between process conditions, biological responses, and scale-dependent behavior.

### 7.2 Reliable and reproducible data sources

Reliable data sources are essential for translating bioprocess knowledge into structured datasets. Microalgal bioprocess data originate from diverse sources, including offline assays, online sensors, at-line measurements, optical and spectroscopic methods, imaging systems, omics platforms, operational logs, literature datasets, and pilot-scale production records (Porras Reyes et al., 2024; Freitas et al., 2025; Yusof et al., 2025).

None of the data sources alone can fully represent microalgal processes, so a good dataset must combine complementary measurements with operational metadata, calibration information, and uncertainty descriptors. Offline assays provide reference labels; online sensors provide temporal structure; imaging and spectroscopic techniques provide biological proxies; pilot-scale records provide scale-dependent variability. However, these resources become truly useful for predictive modeling only when linked to strain identity, reactor configuration, medium composition, operating conditions, sampling frequency, analytical procedures, and calibration status.

Reproducibility should be treated as a data requirement rather than as a reporting detail. For microalgal biofuel systems, each measurement should be traceable to strain identity, medium composition, reactor configuration, scale, illumination strategy, operating mode, sampling protocol, analytical method, and calibration status. Without this context, datasets may support local correlations but fail to represent transferable biological or process relationships (Syed et al., 2024; Yang et al., 2024).

### 7.3 Non-measurable variables and inherent uncertainty

Many variables relevant to microalgal biofuel production cannot be directly measured in real time. Physiological state, intracellular lipid dynamics, cellular stress, contamination risk, and future productivity are often inferred from indirect measurements or proxy variables, posing a major challenge for biology-aware data requirements (Porras Reyes et al., 2024; Barajas-Solano et al., 2026).

Scale-up further complicates this problem because nominal operating conditions do not necessarily represent an effective cellular environment. In larger systems, cells experience heterogeneous light exposure, light-dark cycling, gas transfer limitations, hydrodynamic variability, nutrient gradients, optical fouling, and outdoor fluctuations. Average measurements can mask local conditions that strongly influence photosynthesis, growth, stress, and lipid accumulation (Barajas-Solano et al., 2026).

Software sensors provide a practical approach to estimating biological variables that cannot be measured directly. By combining measurable signals with mechanistic or data-driven models, they estimate latent biological states relevant to productivity, stress, and lipid accumulation, although their assumptions and uncertainty must be carefully validated (Sokolov et al., 2021; Narayanan et al., 2023).

Table 4 summarizes the main latent or difficult-to-quantify variables relevant to process monitoring and digital twin development in microalgal cultivation. These variables cannot be measured directly in real time because their measurement is either technically complex or prohibitively expensive. The data are generally obtained through indirect sampling, software sensors, spectroscopic signals, or model-based estimators. Many of these variables are essential for enhancing process observability, state estimation, and predictive control within microalgae-based biofuel production systems (Sokolov et al., 2021; Narayanan et al., 2023; Barajas-Solano et al., 2026).

**TABLE 4:** Conceptual synthesis of latent or difficult-to-measure variables relevant to microalgal biofuel systems.

Latent or difficult-to-measure variable	Possible proxy or estimator	Uncertainty introduced	Why it matters for biofuels
Physiological state	Chlorophyll fluorescence, pigment profile, growth rate, transcriptomic or metabolomic indicators	A proxy may capture only one dimension of cellular state	Determines whether the culture is growing, stressed, or shifting metabolism
Intracellular lipid accumulation	Nile Red staining, spectroscopic signatures, offline extraction, ML estimators	Calibration depends on species, cell walls, staining conditions, and matrix effects	Directly linked to biodiesel potential and harvest strategy
Cellular stress	Fluorescence changes, ROS proxies, viability, growth deviation	Stress can be multifactorial and not uniquely identifiable from one signal	Stress may increase lipids but reduce biomass and stability
Local light exposure	Irradiance, optical density, reactor geometry, light attenuation models	Incident light does not equal experienced light at the cell level	Controls photosynthesis, photoinhibition, and self-shading
CO_2_ uptake and carbon availability	CO_2_ inlet/outlet, pH, dissolved inorganic carbon estimates, gas transfer models	Carbon chemistry depends on pH, temperature, and mixing	Affects biomass productivity and pH stability
Contamination risk	Microscopy, imaging, anomaly detection	Early contamination may remain visually or analytically subtle	Important for open systems and outdoor cultivation
Future productivity	Biomass trajectory, nutrient status, lipid proxy, software sensor prediction	Prediction depends on model validity and process stability	Supports timing decisions and productivity optimization

This implies that datasets should clearly distinguish directly measured variables from inferred proxies, while also documenting the underlying assumptions, calibration procedures, and uncertainty estimates. Otherwise, in subsequent modeling stages, the inferred states may be treated as direct observations.

Because some biological states cannot be measured directly, the adoption of AI and ML methods that leverage indirect measurements, uncover hidden structures, and predict latent variables in real time is driven. The value of these techniques lies not only in predictive performance but also in improving process observability and facilitating decision-making that accounts for biological factors. Table 5 compares the main AI/ML techniques used to date in the engineering of microalgae-based biofuel production (Coşgun et al., 2023; Chong et al., 2024; Imamoglu, 2024; Syed et al., 2024; Yang et al., 2024; Riezzo et al., 2025). The reported performance metrics were validated against experimental measurements of biomass, lipid content, chlorophyll-related variables, nutrient concentrations, and biochemical state indicators described in the original studies (Sokolov et al., 2021; Narayanan et al., 2023).

**TABLE 5:** Comparative assessment of AI/ML approaches for biology-aware digital twins in microalgal biofuel systems.

AI/ML approach	Primary application	Representative performance^a^	Main scale-up limitation	Validation level	DTs relevance
ANN/MLP	Biomass and lipid prediction	High predictive accuracy reported	Limited transferability	Laboratory-scale	Local prediction
SVR/SVM	Soft sensing and classification	SVR: *R*^2^ = 0.781; RMSE = 0.723; SVM accuracy up to 97.63%	Sensitive to tuning; limited extrapolation	Laboratory-scale	Soft sensing
RNN/LSTM	Biomass forecasting	*R*^2^ = 0.91; RMSE = 0.061 (28,741 records)	Large data requirements	Laboratory-scale	State estimation
CNN and multimodal deep learning	Imaging and lipid estimation	Accuracy up to 97.86%; lipid prediction *R*^2^ = 0.9548	High data demand; protocol dependent	Laboratory-scale	Latent-state estimation
Tree-based ensembles (RF/XGBoost)	Feature selection and process modeling	XGBoost overall *R*^2^ = 0.900	Limited cultivation-scale validation	Literature datasets	Process prediction
Evolutionary optimization (GA)	Process optimization	Optimization-based (non-predictive)	Requires predictive coupling	Laboratory-scale	Decision support
Hybrid mechanistic–ML + transfer learning	State estimation and scale-up	Validation MAPE: 20.1% (biomass), 11.8% (nitrate), 25.2% (lutein)	Calibration and uncertainty management	Laboratory-scale DTs	Biology-aware DTs

^a^Representative performance metrics are illustrative examples from individual studies and should not be interpreted as direct comparisons among AI/ML approaches. ANN, Artificial Neural Network; DTs, Digital Twins; MLP, Multilayer Perceptron; SVR, Support Vector Regression; SVM, Support Vector Machine; RNN, Recurrent Neural Network; LSTM, Long Short-Term Memory; CNN, Convolutional Neural Network; RF, Random Forest; XGBoost, eXtreme Gradient Boosting; GA, Genetic Algorithm; RMSE, Root Mean Square Error; MAPE, Mean Absolute Percentage Error.

## 8 Data-centric digital twins for microalgal biofuels: realistic definition and scope

Once biological and process information has been converted into reliable, reproducible, and model-ready datasets, the next challenge is determining how to integrate those data into computational architectures capable of prediction, scenario analysis, uncertainty management, and operational decision support (Riezzo et al., 2025; Barajas-Solano et al., 2026).

Digital twins have gained considerable attention in industrial digitalization (Tao et al., 2019; Fuller et al., 2020). However, the direct transfer of this concept into biological systems is not straightforward. Microalgal biofuel systems differ fundamentally from conventional industrial systems because they are living, adaptive, nonlinear, partially observable, and highly sensitive to environmental variability (Bernard, 2011; Riezzo et al., 2025).

For this reason, digital twins in microalgal biofuels should not be interpreted as exact virtual replicas of cultivation systems. Such a definition exceeds the current capabilities of sensing technologies, mechanistic understanding, and biological modeling. A more realistic definition is that of a data-centric and biology-aware computational framework capable of integrating process measurements, biological indicators, mechanistic knowledge, uncertainty estimation, predictive analytics, and decision-support mechanisms (Barajas-Solano et al., 2026).

In some studies, almost any predictive model, dashboard, or machine-learning workflow is labeled as a digital twin (Fuller et al., 2020). This broad use weakens the concept and creates confusion regarding the maturity level of current systems. A neural network, regression model, or optimization algorithm by itself is not necessarily a digital twin. A realistic digital twin should include at least four elements: a connection to real or periodically updated process data, a representation of biological or process states, predictive or simulation capabilities, and some level of decision support or operational reasoning.

Consequently, the realistic near-term role of digital twins in microalgal biofuel systems is not fully autonomous cultivation management, but rather uncertainty-aware decision support. Their practical value comes from integrating biological knowledge, process understanding, operational constraints, predictive analytics, and uncertainty estimation into frameworks that support monitoring, optimization, experimental planning, scale-up, and risk assessment (Riezzo et al., 2025).

As the concept matures, it is useful to distinguish between different levels of computational maturity in microalgal systems. Many current systems are described as digital twins despite lacking features such as real-time synchronization, uncertainty handling, or operational decision support (Fuller et al., 2020).

Table 6 summarizes representative computational systems and their maturity characteristics reported in the literature.

**TABLE 6:** Maturity levels of computational systems in microalgal biofuel applications.

System type	Main characteristics	Real-time connection	Predictive capability	Biological representation	Decision-support capability
Dashboard platform	Visualization of process variables	Partial	Minimal	Weak	Information display
Standalone ML predictor	Data-driven prediction	Offline	High within the training domain	Limited	Limited
Process simulator	Mechanistic process model	Offline	Moderate	Moderate to strong	Scenario analysis
Soft sensor system	Latent-state estimation from measurable signals	Near real-time	Moderate	Moderate	Monitoring
Hybrid digital twin	Integration of process knowledge, operational data, ML, and uncertainty	Continuous/real-time	High	Strong	Decision support

As shown in Table 6, most current microalgal systems lie between standalone predictive tools and partial hybrid frameworks. Fully integrated digital twins capable of autonomous operation remain uncommon because of the biological and operational complexity of microalgal cultivation systems.

### 8.1 Predictive modeling and uncertainty management in microalgal systems

Predictive modeling is a core component of data-centric digital twins. In microalgal biofuel systems, predictive models are commonly used to estimate biomass productivity, lipid accumulation, stress conditions, and overall process efficiency.

Machine-learning approaches provide a flexible framework for modeling the nonlinear relationships that characterize microalgal cultivation systems, including growth, lipid accumulation, nutrient utilization, and stress responses. However, their performance often depends on the quality and representativeness of the training data. Models developed under controlled laboratory conditions may achieve high predictive accuracy yet fail when transferred to different strains, cultivation systems, environmental conditions, or operational scales. Predictive performance should be evaluated not only through statistical metrics but also through biological realism, transferability, and operational reliability (Coşgun et al., 2023; Imamoglu, 2024).

#### 8.1.1 Model validation, robustness, and generalization

A digital twin is only as reliable as its predictive layer. Validation should not be treated as a secondary methodological detail. In microalgal biofuel systems, robustness and generalization remain challenging because biological behavior changes significantly across strains, environmental conditions, cultivation modes, and operational scales.

Mechanistic models preserve interpretability and process consistency but may oversimplify biological variability. In contrast, ML models are more flexible but often struggle to generalize beyond the conditions represented in their training data. This challenge is particularly evident during scale-up, where gradients, fluctuating irradiance, heterogeneous hydrodynamics, and varying gas-transfer conditions create environments that differ substantially from laboratory cultures. Recent studies emphasize hybrid modeling, transfer learning, and uncertainty-aware approaches as strategies to improve model robustness and cross-scale applicability (Herrera-Ruiz et al., 2025; Riezzo et al., 2025).

For operational digital twins, the performance of internal datasets is insufficient. Validation should include independent experimental validation, cross-strain testing, cross-scale transferability, sensitivity analysis, uncertainty estimation, resilience to noisy or incomplete data, and a clearly defined domain of applicability. Without these components, digital twins risk becoming highly optimized laboratory predictors with limited industrial relevance.

#### 8.1.2 Uncertainty handling and limits of extrapolation

Uncertainty management remains one of the most underdeveloped components of current digital-twin research in microalgal biofuel systems.

Biological and measurement uncertainty are unavoidable in microalgal cultivation systems. Microalgae continuously respond to environmental fluctuations, while sensors, sampling procedures, and analytical methods introduce additional sources of variability. Decision-support systems require not only predictions but also estimates of confidence and operational risk. This becomes particularly important during scale-up, where fluctuating irradiance, contamination events, temperature shifts, hydrodynamic variability, and gas-transfer limitations may place the system outside the conditions represented in the training data. Herrera-Ruiz et al. (2025) emphasize that nonparametric models become especially vulnerable outside their interpolation domain. Riezzo et al. (2025) also show that transfer-learning-based digital twins still requires explicit uncertainty quantification when adapting to new strains or operating conditions.

Uncertainty should not be treated as a statistical afterthought. In biologically realistic digital twins, uncertainty should be part of the system’s operational language. A biologically plausible but uncertainty-aware prediction is often more useful than a highly confident prediction without biological support.

### 8.2 Hybrid modeling and data integration in digital twins

#### 8.2.1 Integration of heterogeneous experimental data

Recent studies increasingly combine CFD, process simulation, machine learning, and optimization frameworks, highlighting the need to integrate biological and engineering information within unified decision-support architectures (González-Delgado et al., 2025).

A major weakness in the current literature is that many studies describe algorithms but provide limited detail on data synchronization, preprocessing, harmonization, metadata integration, or uncertainty propagation. In practice, data preparation often influences model quality more than the choice of algorithm itself.

#### 8.2.2 Hybrid models combining process knowledge and data-driven approaches

Hybrid modeling is increasingly recognized as an effective approach for developing realistic digital twins in microalgal biofuel systems. Mechanistic models provide interpretability and process consistency but often oversimplify biological dynamics, whereas data-driven models offer greater flexibility at the expense of biological transparency. Hybrid models seek to balance these strengths by integrating mechanistic knowledge with adaptive learning approaches.

Recent studies identify hybrid modeling as a practical compromise between white-box and black-box approaches, particularly for biological systems characterized by partial observability and incomplete mechanistic understanding (Herrera-Ruiz et al., 2025).

Transfer-learning-based hybrid frameworks have also been proposed to transfer knowledge between related strains while updating uncertain components with new experimental data, thereby reducing experimental burden and improving adaptability (Riezzo et al., 2025). Hybrid modeling should not be viewed as a universal solution, but rather as a structured strategy for balancing knowledge and data.

As summarized in Table 7, no single modeling strategy fully addresses the biological and operational complexity of microalgal biofuel systems (González-Delgado et al., 2025; Herrera-Ruiz et al., 2025; Riezzo et al., 2025).

**TABLE 7:** Literature-based comparison of modeling strategies for microalgal digital twins.

Modeling strategy	Main strengths	Main weaknesses	Main risk	Suitability for digital twins
Pure mechanistic models	Interpretable and physically grounded	Limited representation of biological complexity	Oversimplification	Structural backbone
Pure machine-learning models	Flexible and predictive	Large representative datasets	Poor extrapolation	Local prediction
Statistical soft sensors	Latent-state estimation	Calibration dependent	Transferability issues	Monitoring
Hybrid models	Mechanistic–data integration	More complex implementation	Accumulated uncertainty	Most promising
Transfer-learning frameworks	Knowledge transfer	Sensitive to domain shift	Hidden bias transfer	Emerging strategy

The most promising direction is the development of hybrid, uncertainty-aware, and biology-informed frameworks that integrate mechanistic knowledge with adaptive, data-driven components.

#### 8.2.3 Soft sensors and latent biological states

Soft sensors play a central role in expanding the observable state of microalgal cultivation systems. Many biologically important variables cannot be measured directly in real time, including intracellular lipid accumulation, physiological stress, contamination risk, local irradiance exposure, or future productivity.

Soft sensors estimate these latent variables from measurable signals such as fluorescence, absorbance, dissolved oxygen, optical density, spectral fingerprints, or image-derived features (Porras Reyes et al., 2024). Relationships between observable signals and latent biological variables are often strain-dependent, stress-dependent, and scale-dependent. A fluorescence-based estimator calibrated for one species may fail under different physiological conditions or cultivation environments (González-Delgado et al., 2025).

Soft sensors should be treated as uncertainty-aware estimation modules rather than direct substitutes for biological measurements.

### 8.3 Interpretability and decision-support layers

#### 8.3.1 Interpretability and explainable digital twins

Interpretability is increasingly important as digital twins incorporates more complex AI models. Explainability methods can help identify which variables influence predictions and improve user confidence. However, interpretability should not be confused with causality, since highly influential variables may correlate with unmeasured drivers. Explainable digital twins should combine transparent modeling strategies with domain expertise to support biologically meaningful decision-making (Lundberg and Lee, 2017).

#### 8.3.2 Decision-support layers without technological overclaiming

The most realistic near-term role of digital twins is decision support rather than fully autonomous control. Digital twins can assist scenario analysis, nutrient management, contamination detection, harvest optimization, operational risk assessment, and multi-objective optimization.

Fully autonomous cultivation remains unrealistic because it requires reliable sensing, adaptive control, fault tolerance, and robust cross-scale validation that are not yet routinely available (Sangregorio-Soto et al., 2026).

Consequently, a human-in-the-loop paradigm remains the most realistic near-term framework for microalgal biofuel applications. In this context, digital twins serve as a transparent computational assistant that integrates biological knowledge, uncertainty estimation, predictive analytics, and process understanding into operational guidance.

Table 8 summarizes the major unresolved challenges repeatedly identified across recent studies on digital twins, soft sensing, uncertainty-aware modeling, and AI-assisted microalgal bioprocesses (Syed et al., 2024; Herrera-Ruiz et al., 2025; Sangregorio-Soto et al., 2026).

**TABLE 8:** Main unresolved challenges reported for digital twins in microalgal biofuel systems.

Challenge	Why it matters	Current limitations in the literature	Future direction
Dataset standardization	Enables reproducibility and transferability	Experimental heterogeneity remains high	Common reporting standards
Cross-scale transferability	Laboratory models often fail at larger scales	Weak external validation	Pilot and outdoor benchmarking
Uncertainty quantification	Needed for reliable operational decisions	Deterministic predictions dominate	Bayesian and probabilistic approaches
Biological interpretability	Prevents black-box decision-making	Accuracy is often prioritized over explanation	Explainable hybrid models
Latent biological states	Key variables remain difficult to measure directly	Weak biological sensing	Advanced soft sensors
Synthetic-data validation	Artificial data may violate biological constraints	Statistical validation dominates	Biology-aware validation
Autonomous operation	Full automation remains unrealistic	Limited robustness and fault tolerance	Human-in-the-loop systems

Current evidence suggests that digital twins for microalgal biofuels should be viewed as evolving, multi-layered systems rather than fully mature technologies. Their future development will depend less on isolated algorithmic advances and more on the creation of coherent biological data ecosystems that integrate experimentation, process engineering, soft sensing, uncertainty-aware modeling, hybrid modeling, and decision support. In this context, the value of digital twins lies not in replacing biological expertise but in supporting it through transparent, biologically informed, and operationally realistic computational frameworks (Porras Reyes et al., 2024).

## 9 Automation and intelligent control in microalgal cultivation

The integration of sensors, actuators, and control systems within a mechatronic architecture transforms a simple container into an automated photobioreactor. An efficient design must strike a balance among light utilization, adequate mass transfer between gas and liquid phases, and reduced energy consumption (Chanquia et al., 2022).

### 9.1 Control architectures

The control system forms the core of the automated photobioreactor. The choice between PLCs and microcontrollers depends primarily on the level of robustness and flexibility required (Wang et al., 2020).

PLCs are widely used in industrial applications due to their reliability, modularity, and built-in safety features. They are particularly well-suited for commercial facilities where operational continuity is a priority. In contrast, platforms such as ESP32 or Raspberry Pi are common in research and experimental prototypes due to their low cost and ease of integration with IoT technologies, artificial intelligence, and cloud data processing (Baicu et al., 2024). Given the biological complexity of microalgae, algorithmic architectures have evolved to counteract disturbances and uncertainties (Wang et al., 2020; Sangregorio-Soto et al., 2026).

Advanced control architectures such as ADRC and MRAC have been proposed to improve disturbance rejection, adaptive response, and operational stability under uncertain biological and environmental conditions (Sangregorio-Soto et al., 2026).

### 9.2 Advanced control strategies and artificial intelligence

Modern photobioreactor automation has evolved beyond traditional feedback systems, incorporating advanced strategies to handle the nonlinear nature of biological processes (Ryalat et al., 2024).

#### 9.2.1 PID control

The PID controller remains a very important part of industrial automation. However, due to process inertia and nonlinear variables such as pH, conventional PID control may be insufficient for microalgal cultivation systems (Otálora et al., 2023). To overcome these limitations, fractional-order PID (FOPID) controllers are proposed, which provide additional degrees of freedom, faster response, and the iso-damping property, resulting in greater phase margin and larger variations in system parameters (Shah and Agashe, 2016; Jamil et al., 2022). In addition to the classical PID tuning parameters, the FOPID method introduces two additional tuning parameters, thereby providing better control for nonlinear, time-varying systems than traditional PID controllers. Recent studies have reported enhanced closed-loop performance, including faster settling time, smaller overshoot, smaller steady-state error, stronger disturbance rejection, and more robust pH tracking under process disturbances and model uncertainty (Bashishtha et al., 2025). These features make FOPID well suited for controlling microalgal cultivation systems, which exhibit nonlinear, time-varying, and memory-dependent behavior. Some systems also use self-tuning algorithms that dynamically adjust control parameters in response to environmental changes (Ouyang et al., 2023; Caparroz et al., 2025).

#### 9.2.2 Machine learning and predictive analytics

AI is becoming a major trend in PBRs automation. Machine learning algorithms enable for simultaneous analysis of data from physical sensors, images, and historical variables to optimize growth and productivity. These approaches support predictive control of cultivation conditions, nutrient dosing, mixing, and energy consumption while improving process efficiency and operational control (Lim et al., 2022; Sangregorio-Soto et al., 2026).

The evolution of microalgae cultivation systems toward automated, mechatronics-based platforms is essential for improving the economic viability and scalability of this technology. However, significant challenges remain, particularly regarding sensor biofouling, the complexity of biological systems, and the need for more robust predictive models. In this context, the combination of physical modeling, artificial intelligence, and industrial automation is becoming a promising direction for future development.

## 10 Techno-economic and environmental implications of microalgal scale-up

Techno-economic assessment (TEA) is commonly used to evaluate the economics of microalgal biofuel production routes and identify key cost components that hinder commercial implementation. Cultivation, harvesting, dewatering, extraction, and downstream processing are still major cost contributors. TEA provides a useful framework for prioritizing research and development efforts and supporting scale-up decisions (Zimmermann et al., 2020). Although both perspectives should be included in the evaluation of microalgal biodiesel systems, relatively few studies integrate these methodologies. This limitation is largely due to the scarcity of large-scale production data and the reliance on laboratory- or pilot-scale extrapolations (Nogales-Delgado, 2025).

### 10.1 LCA and TEA applied to algal biodiesel production

LCA and TEA studies evaluate the potential of algal biodiesel as an alternative to fossil and first-generation biofuels by assessing environmental performance, energy efficiency, resource consumption, and economic feasibility (Mu et al., 2020).

Greenhouse emissions: Several reports indicate that algal biodiesels reduce greenhouse gas emissions by up to 60% compared with conventional diesel. Nevertheless, current technologies for producing algal biodiesel have limitations, and to significantly reduce greenhouse gas emissions, the energy used in the production process must be decarbonized. Unfortunately, a meta-analysis found that biodiesels may not outperform conventional diesel, so the environmental advantages of algal biodiesel remain uncertain (Garcia et al., 2020; Bradley et al., 2023).

Energy balance: Energy demand depends not only on the production system but also on the configuration of the PBRs or RWPs. Simulations of energy demand show that the process from biomass cultivation through dewatering, lipid extraction, and transesterification is energy-losing, even in optimistic scenarios. Therefore, the entire process should be optimized to avoid energy losses, and, in that case, biodiesel could become competitive with fossil diesel (Mu et al., 2020).

Nutrient and water management remain major sustainability concerns. Strategies such as nutrient recycling, anaerobic digestion, and wastewater integration can reduce fertilizer demand and freshwater consumption while improving overall process sustainability (Bradley et al., 2023).

Microalgal biodiesel generally exhibits a lower land footprint than conventional oilseed crops. However, large-scale deployment still requires extensive cultivation infrastructure, and trade-offs between land use, biomass productivity, and economic cost remain important when comparing open ponds and PBRs (Sander and Murthy, 2010).

Biorefinery and integration strategies: Producing multiple value-added coproducts improves the economic feasibility of algal biodiesel production (Herrera et al., 2021).

TEA studies consistently identify cultivation, harvesting, dewatering, extraction, and conversion processes as the major cost drivers in microalgal biodiesel production. Photobioreactors provide higher productivity and process control but require greater capital investment, whereas open ponds offer lower costs but are more susceptible to contamination and lower biomass density (Delrue et al., 2012; Xin et al., 2016).

Harvesting and dewatering remain major bottlenecks, as they account for substantial energy consumption and operating costs. Although technologies such as centrifugation provide high recovery efficiencies, their large-scale implementation remains economically challenging, motivating the development of lower-energy alternatives. Extraction and transesterification also influence overall process economics because conversion efficiency directly affects biodiesel yield. Improvements in lipid recovery and catalytic conversion remain priorities for process optimization. Sensitivity analyses consistently identify biomass productivity, lipid content, cultivation system design, harvesting efficiency, nutrient recycling, and plant capacity as the variables that have the greatest influence on biodiesel production costs (Lee et al., 2019).

The transition of microalgal biofuels to commercial production remains limited by the integration of biological, engineering, and computational challenges. Its industrial application will increasingly depend on AI-enabled cultivation systems, ML models, and digital twins. These methodologies still face limitations, such as data availability, dataset standardization, sensor reliability, uncertainty quantification, and long-term validation in an industrially relevant environment. Future progress will depend on closer integration of biological engineering, process engineering, standardized data infrastructures, and computational modeling (Syed et al., 2024; Herrera-Ruiz et al., 2025).

## 11 Roadmap 2026–2036 for industrially relevant microalgal biofuels

The next decade will be critical in determining whether microalgal biofuels can move from laboratory-scale research to economically viable industrial applications. Future progress will require integrated solutions that simultaneously address biological robustness, process efficiency, resource utilization, automation, and sustainability throughout scale-up (Geng et al., 2025).

Priority should be given to robust industrial strains, resource-efficient cultivation systems, and circular biorefinery strategies that integrate wastewater treatment, carbon capture, nutrient recycling, and biomass valorization to improve both economic and environmental performance (Geng et al., 2025). Biology-aware digital twins have the potential to integrate physiological, environmental, operational, and economic information into predictive frameworks that support scale-up and industrial operation (Porras Reyes et al., 2024).

Industrial viability will depend not on a single technological breakthrough, but on the integration of systems biology, process engineering, AI, digital twins, and circular biorefinery strategies. Future technologies should be evaluated using integrated techno-economic and life-cycle assessment frameworks to support sustainable commercial deployment (Geng et al., 2025).

## 12 Conclusion

Microalgal biofuels have considerable potential, but moving from laboratory studies to industrial production remains a major challenge. Improving a single process or technology is not enough to achieve industrial-scale production. Successful scale-up requires integrating biological knowledge with reactor design, process monitoring, automation, and computational tools to better understand and control the complexity of microalgal cultivation.

Biology should guide the development of engineering and digital technologies rather than be treated as another process variable. Biology-aware, data-centric digital twins provide a framework for integrating biological knowledge, experimental data, mechanistic models, and artificial intelligence to support monitoring, prediction, and decision-making. Their value lies in helping researchers and operators interpret complex biological systems, not in replacing experimental work or human expertise. This approach shifts the focus from optimizing individual processes to managing microalgal cultivation as an integrated biological and engineering system supported by reliable data and computational models.

The future of microalgal biofuels will depend on integrating biology, engineering, and data science to build reliable and sustainable production systems. Achieving this objective will be essential to improving process robustness, facilitating industrial scale-up, and strengthening the role of microalgal biofuels within the circular bioeconomy.

## References

[B1] Abdelfattah A. Ali S. S. Ramadan H. El-Aswar E. I. Eltawab R. Ho S.-H. et al. (2023). Microalgae-based wastewater treatment: Mechanisms, challenges, recent advances, and future prospects. *Environ. Sci. Ecotechnol.* 13:100205. 10.1016/j.ese.2022.100205 36247722 PMC9557874

[B2] Alishah Aratboni H. Rafiei N. Garcia-Granados R. Alemzadeh A. Morones-Ramírez J. R. (2019). Biomass and lipid induction strategies in microalgae for biofuel production and other applications. *Microb. Cell Fact.* 18:178. 10.1186/s12934-019-1228-4 31638987 PMC6805540

[B3] Avinash A. Sasikumar P. Pugazhendhi A. (2020). Analysis of the limiting factors for large scale microalgal cultivation: A promising future for renewable and sustainable biofuel industry. *Renew. Sustain. Energy Rev.* 134:;110250. 10.1016/j.rser.2020.110250

[B4] Aziz M. M. A. Kassim K. A. Shokravi Z. Jakarni F. M. Liu H. Y. Zaini N. et al. (2020). Two-stage cultivation strategy for simultaneous increases in growth rate and lipid content of microalgae: A review. *Renew. Sustain. Energy Rev.* 119:109621. 10.1016/j.rser.2019.109621

[B5] Baicu L. M. Andrei M. Ifrim G. A. Dimitrievici L. T. (2024). Embedded IoT design for bioreactor sensor integration. *Sensors* 24:6587. 10.3390/s24206587 39460068 PMC11510879

[B6] Barajas-Solano A. F. Zuorro A. Lavecchia R. García-Martínez J. B. Contreras-Ropero J. E. (2026). Bioprocess design and optimization for pharmaceutical production using microalgae and cyanobacteria. *Processes* 14:1141. 10.3390/pr14071141

[B7] Bashishtha T. K. Singh V. P. Yadav U. K. Sahu U. K. (2025). Fractional-order PID controllers and applications: A comprehensive survey. *Annu. Rev. Control* 60:101013. 10.1016/j.arcontrol.2025.101013

[B8] Béchet Q. Shilton A. Guieysse B. (2016). Maximizing productivity and reducing environmental impacts of full-scale algal production through optimization of open pond depth and hydraulic retention time. *Environ. Sci. Technol.* 50 4102–4110. 10.1021/acs.est.5b05412 26928398

[B9] Bernard O. (2011). Hurdles and challenges for modelling and control of microalgae for CO_2_ mitigation and biofuel production. *J. Process Control* 21 1378–1389. 10.1016/j.jprocont.2011.07.012

[B10] Bharti R. K. Singh A. Dhar D. W. Kaushik A. (2022). “Biological Carbon Dioxide Sequestration by Microalgae for Biofuel and Biomaterials Production,” in *Biomass, Biofuels, Biochemicals*, eds Thakur I. S. Pandey A. Ngo H. H. Soccol C. R. Larroche C. (Oxford: Elsevier), 137–153.

[B11] Bradley T. Rajaeifar M. A. Kenny A. Hainsworth C. del Pino V. del Valle et al. (2023). Life cycle assessment of microalgae-derived biodiesel. *Int. J. Life Cycle Assess.* 28 590–609. 10.1007/s11367-023-02140-6

[B12] Brennan L. Owende P. (2010). Biofuels from microalgae—A review of technologies for production, processing, and extractions of biofuels and co-products. *Renew. Sustain. Energy Rev.* 14 557–577. 10.1016/j.rser.2009.10.009

[B13] Brindhadevi K. Mathimani T. Rene E. R. Shanmugam S. Chi N. T. L. Pugazhendhi A. (2021). Impact of cultivation conditions on the biomass and lipid in microalgae with an emphasis on biodiesel. *Fuel* 284:119058. 10.1016/j.fuel.2020.119058

[B14] Caparroz M. Soltesz K. Hägglund T. Guzmán J. L. Berenguel M. (2025). A new approach to relay-based autotuning PID controllers and their evaluation in pH control of industrial photobioreactors. *Control Eng. Pract.* 164:106520. 10.1016/j.conengprac.2025.106520

[B15] Carlozzi P. (2003). Dilution of solar radiation through “culture” lamination in photobioreactor rows facing south-north: A way to improve the efficiency of light utilization by cyanobacteria (*Arthrospira platensis*). *Biotechnol. Bioeng.* 81 305–315. 10.1002/bit.10478 12474253

[B16] Carvalho A. P. Meireles L. A. Malcata F. X. (2006). Microalgal reactors: A review of enclosed system designs and performances. *Biotechnol. Prog.* 22 1490–1506. 10.1021/bp060065r 17137294

[B17] Chan D. Chien J.-C. Axpe E. Blankemeier L. Baker S. W. Swaminathan S. et al. (2022). Combinatorial polyacrylamide hydrogels for preventing biofouling on implantable biosensors. *Adv. Mater.* 34:2109764. 10.1002/adma.202109764 35390209 PMC9793805

[B18] Chanquia S. N. Vernet G. Kara S. (2022). Photobioreactors for cultivation and synthesis: Specifications, challenges, and perspectives. *Eng. Life Sci.* 22 712–724. 10.1002/elsc.202100070 36514531 PMC9731602

[B19] Cheirsilp B. Maneechote W. Srinuanpan S. Angelidaki I. (2023). Microalgae as tools for bio-circular-green economy: Zero-waste approaches for sustainable production and biorefineries of microalgal biomass. *Bioresour. Technol.* 387:129620. 10.1016/j.biortech.2023.129620 37544540

[B20] Chen B.-L. Mhuantong W. Ho S.-H. Chang J.-S. Zhao X.-Q. Bai F.-W. (2020). Genome sequencing, assembly, and annotation of the self-flocculating microalga *Scenedesmus obliquus* AS-6-11. *BMC Genom.* 21:743. 10.1186/s12864-020-07142-4 33109102 PMC7590803

[B21] Chen J. Li Y. Anderson A. Liu S. Dai L. Wang L. et al. (2025). Evaluation and prevention of biofilm formation on stainless steel sensor mesh for effective biofouling mitigation. *Algae Environ.* 1:4. 10.53941/algaeenviron.2025.100004

[B22] Chew K. W. Yap J. Y. Show P. L. Suan N. H. Juan J. C. Ling T. C. et al. (2017). Microalgae biorefinery: High value products perspectives. *Bioresour. Technol.* 229 53–62. 10.1016/j.biortech.2017.01.006 28107722

[B23] Chiaramonti D. Prussi M. Casini D. Tredici M. R. Rodolfi L. Bassi N. et al. (2013). Review of energy balance in raceway ponds for microalgae cultivation: Re-thinking a traditional system is possible. *Appl. Energy* 102 101–111. 10.1016/j.apenergy.2012.07.040

[B24] Chisti Y. (2007). Biodiesel from microalgae. *Biotechnol. Adv.* 25 294–306. 10.1016/j.biotechadv.2007.02.001 17350212

[B25] Chong J. W. R. Khoo K. S. Chew K. W. Ting H.-Y. Iwamoto K. Ruan R. et al. (2024). Artificial intelligence-driven microalgae autotrophic batch cultivation: A comparative study of machine and deep learning-based image classification models. *Algal Res.* 79:103400. 10.1016/j.algal.2024.103400

[B26] Correa-Aguado H. C. Cerrillo-Rojas G. V. Rocha-Uribe A. Soria-Guerra R. E. Morales-Domínguez J. F. (2021). Benzyl amino purine and gibberellic acid coupled to nitrogen-limited stress induce fatty acids, biomass accumulation, and gene expression in *Scenedesmus obliquus*. *Phyton-Int. J. Exp. Bot.* 90 515–531. 10.32604/phyton.2021.013619

[B27] Coşgun A. Günay M. E. Yıldırım R. (2023). Machine learning for algal biofuels: A critical review and perspective for the future. *Green Chem.* 25 3354–3373. 10.1039/D3GC00389B

[B28] Cotta F. C. Amaral R. Bacellar F. L. Correia D. Asadi K. Rocha P. R. F. (2025). A 3D porous electrode for real-time monitoring of microalgal growth and exopolysaccharides yields using Electrochemical Impedance Spectroscopy. *Biosens. Bioelectron.* 277:117260. 10.1016/j.bios.2025.117260 39983293

[B29] Craig R. J. Hasan A. R. Ness R. W. Keightley P. D. (2021). Comparative genomics of *Chlamydomonas*. *Plant Cell* 33 1016–1041. 10.1093/plcell/koab026 33793842 PMC8226300

[B30] de Godos I. Mendoza J. L. Acién F. G. Molina E. Banks C. J. Heaven S. et al. (2014). Evaluation of carbon dioxide mass transfer in raceway reactors for microalgae culture using flue gases. *Bioresour. Technol.* 153 307–314. 10.1016/j.biortech.2013.11.087 24374031

[B31] de Paula Pereira A. S. A. Silva T. A. Magalhães I. B. Ferreira J. Braga M. Q. Lorentz J. F. et al. (2024). Biocompounds from wastewater-grown microalgae: A review of emerging cultivation and harvesting technologies. *Sci. Total Environ.* 920:170918. 10.1016/j.scitotenv.2024.170918 38354809

[B32] Dȩbowski M. Kazimierowicz J. Zieliński M. (2025). Multi-Sensing monitoring of the microalgae biomass cultivation systems for biofuels and added value products synthesis—challenges and opportunities. *Appl. Sci.* 15:7324. 10.3390/app15137324

[B33] Dȩbowski M. Świca I. Kazimierowicz J. Zieliński M. (2022). Large scale microalgae biofuel technology—development perspectives in light of the barriers and limitations. *Energies* 16:81. 10.3390/en16010081

[B34] Dȩbowski M. Zieliński M. Kazimierowicz J. Kujawska N. Talbierz S. (2020). Microalgae cultivation technologies as an opportunity for bioenergetic system development—advantages and limitations. *Sustainability* 12:9980. 10.3390/su12239980

[B35] Delgado A. Briciu-Burghina C. Regan F. (2021). Antifouling strategies for sensors used in water monitoring: Review and future perspectives. *Sensors* 21 389. 10.3390/s21020389 33429907 PMC7827029

[B36] Delrue F. Setier P. A. Sahut C. Cournac L. Roubaud A. Peltier G. et al. (2012). An economic, sustainability, and energetic model of biodiesel production from microalgae. *Bioresour. Technol.* 111 191–200. 10.1016/j.biortech.2012.02.020 22366604

[B37] Dey S. Sahu A. Dalvi V. De B. S. Malik A. (2025). Engineering an automated microbubble-assisted hybrid photobioreactor for CO_2_ capture and valorisation to polyhydroxybutyrate in indigenous algal biomass. *Front. Chem. Eng.* 7:1701857. 10.3389/fceng.2025.1701857

[B38] Emamshoushtari M. M. Tavakoli O. Harasek M. Haddadi B. Pajoum Sharitati F. (2026). CFD–experimental assessment of open-tube airlift photobioreactors for CO_2_ capture and nutrient removal. *J. Environ. Chem. Eng.* 14:120533. 10.1016/j.jece.2025.120533

[B39] Ezhumalai G. Arun M. Manavalan A. Rajkumar R. Heese K. (2024). A holistic approach to circular bioeconomy through the sustainable utilization of microalgal biomass for biofuel and other value-added products. *Microb. Ecol.* 87:61. 10.1007/s00248-024-02376-1 38662080 PMC11045622

[B40] Forster P. M. Smith C. Walsh T. Lamb W. F. Lamboll R. Hall B. et al. (2024). Indicators of Global Climate Change 2023: Annual update of key indicators of the state of the climate system and human influence. *Earth Syst. Sci. Data* 16 2625–2658. 10.5194/essd-16-2625-2024

[B41] Freitas G. R. Badenes S. Oliveira R. Martins F. G. (2025). A review of intelligent modeling for microalgae systems: Integrating data mining, machine learning, and hybrid approaches. *Processes* 13:2956. 10.3390/pr13092956

[B42] Fu J. Huang Y. Liao Q. Xia A. Fu Q. Zhu X. (2019). Photo-bioreactor design for microalgae: A review from the aspect of CO_2_ transfer and conversion. *Bioresour. Technol.* 292:121947. 10.1016/j.biortech.2019.121947 31466821

[B43] Fuchs T. Arnold N. D. Garbe D. Deimel S. Lorenzen J. Masri M. et al. (2021). A newly designed automatically controlled, sterilizable flat panel photobioreactor for axenic algae culture. *Front. Bioeng. Biotechnol.* 9:697354. 10.3389/fbioe.2021.697354 34277591 PMC8280782

[B44] Fuller A. Fan Z. Day C. Barlow C. (2020). Digital twin: Enabling technologies, challenges and open research. *IEEE Access* 8 108952–108971. 10.1109/ACCESS.2020.2998358

[B45] Gao Y. Bernard O. Fanesi A. Perré P. Lopes F. (2024). The effect of light intensity on microalgae biofilm structures and physiology under continuous illumination. *Sci. Rep.* 14:1151. 10.1038/s41598-023-50432-6 38212356 PMC10784318

[B46] Garcia R. Figueiredo F. Brandão M. Hegg M. Castanheira É Malça J. et al. (2020). A meta-analysis of the life cycle greenhouse gas balances of microalgae biodiesel. *Int. J. Life Cycle Assess* 25 1737–1748. 10.1007/s11367-020-01780-2

[B47] Gaurav K. Neeti K. Singh R. (2024). Microalgae-based biodiesel production and its challenges and future opportunities: A review. *Green Technol. Sustain.* 2:100060. 10.1016/j.grets.2023.100060

[B48] Geng Y. Shaukat A. Azhar W. Raza Q.-U.-A. Tahir A. Abideen M. Z. U. et al. (2025). Microalgal biorefineries: A systematic review of technological trade-offs and innovation pathways. *Biotechnol. Biofuels Bioprod.* 18:93. 10.1186/s13068-025-02694-7 40817213 PMC12357411

[B49] González-Delgado ÁD. Rojas-Flores S. Alviz-Meza A. (2025). Trends in using microalgae as a green energy source: Conventional, machine learning, and hybrid modeling methods. *Processes* 13:3134. 10.3390/pr13103134

[B50] Grobbelaar J. U. (2012). Microalgae mass culture: The constraints of scaling-up. *J. Appl. Phycol.* 24 315–318. 10.1007/s10811-011-9728-6

[B51] Hajinajaf N. Fallahi A. Eustance E. Sarnaik A. Askari A. Najafi M. et al. (2024). Managing carbon dioxide mass transfer in photobioreactors for enhancing microalgal biomass productivity. *Algal Res.* 80:103506. 10.1016/j.algal.2024.103506

[B52] Hasan M. M. Mofijur M. Uddin M. N. Kabir Z. Badruddin I. A. Khan T. M. Y. (2024). Insights into anaerobic digestion of microalgal biomass for enhanced energy recovery. *Front. Energy Res.* 12:1355686. 10.3389/fenrg.2024.1355686

[B53] Hashmi Z. Idriss I. M. Zaini J. Abu Bakar M. S. Bilad M. R. (2025). Advancements in photobioreactor systems: Optimizing operations for enhanced microalgal growth and bioremediation. *Algal Res.* 91:104282. 10.1016/j.algal.2025.104282

[B54] Havlik I. Beutel S. Scheper T. Reardon K. F. (2022). On-Line monitoring of biological parameters in microalgal bioprocesses using optical methods. *Energies* 15:875. 10.3390/en15030875

[B55] He Z.-M. Cao J.-P. Zhao X.-Y. (2022). Review of biomass agglomeration for fluidized-bed gasification or combustion processes with a focus on the effect of alkali salts. *Energy Fuels* 36 8925–8947. 10.1021/acs.energyfuels.2c01183

[B56] Herrera A. D’Imporzano G. Acién Fernandez F. G. Adani F. (2021). Sustainable production of microalgae in raceways: Nutrients and water management as key factors influencing environmental impacts. *J. Clean. Prod.* 287:125005. 10.1016/j.jclepro.2020.125005

[B57] Herrera-Ruiz J. F. Fontalvo J. Prado-Rubio O. A. (2025). Hybrid modeling for bioprocesses: Architectures, applications, and perspectives. *Eng. Rep.* 7:e70502. 10.1002/eng2.70502

[B58] Hoeniges J. Welch W. Pruvost J. Pilon L. (2022). A novel external reflecting raceway pond design for improved biomass productivity. *Algal Res.* 65:102742. 10.1016/j.algal.2022.102742

[B59] Huang T. Ma S. Wang J. Huang Y. Zhu X. Xia A. et al. (2026). Prediction and optimization of light transmission to enhance microalgae growth in built-in light sources photobioreactor for efficient CO_2_ fixation. *J. Clean. Prod.* 558:148280. 10.1016/j.jclepro.2026.148280

[B60] Huesemann M. Gao S. Edmundson S. Laurens L. M. L. Van Wychen S. Beirne N. et al. (2023). DISCOVR strain pipeline screening – Part II: Winter and summer season areal productivities and biomass compositional shifts in climate-simulation photobioreactor cultures. *Algal Res.* 70:102948. 10.1016/j.algal.2022.102948

[B61] Imamoglu E. (2024). Artificial intelligence and/or machine learning algorithms in microalgae bioprocesses. *Bioengineering* 11:1143. 10.3390/bioengineering11111143 39593803 PMC11592280

[B62] IPCC (2023). *Climate Change 2023: Synthesis Report. Contribution of Working Groups I, II and III to the Sixth Assessment Report of the Intergovernmental Panel on Climate Change*, ed. Romero H. L. J. (Geneva: Intergovernmental Panel on Climate Change).

[B63] Jamil A. A. Tu W. F. Ali S. W. Terriche Y. Guerrero J. M. (2022). Fractional-Order PID controllers for temperature control: A review. *Energies* 15:3800. 10.3390/en15103800

[B64] Jia F. Kacira M. Ogden K. L. (2015). Multi-Wavelength based optical density sensor for autonomous monitoring of microalgae. *Sensors* 15 22234–22248. 10.3390/s150922234 26364640 PMC4610439

[B65] Jorquera O. Kiperstok A. Sales E. A. Embiruçu M. Ghirardi M. L. (2010). Comparative energy life-cycle analyses of microalgal biomass production in open ponds and photobioreactors. *Bioresour. Technol.* 101 1406–1413. 10.1016/j.biortech.2009.09.038 19800784

[B66] Kabir A. Shitu A. Ye Z. Li X. Ma H. Liu G. et al. (2026). Sustainable Recirculating Aquaculture Systems (RAS): Development and challenges. *Water* 18:1093. 10.3390/w18091093

[B67] Khozin-Goldberg I. (2016). “Lipid Metabolism in Microalgae,” in *The Physiology of Microalgae*, eds Borowitzka M. A. Beardall J. Raven J. A. (Cham: Springer International Publishing), 413–484.

[B68] Kong F. Blot C. Liu K. Kim M. Li-Beisson Y. (2024). Advances in algal lipid metabolism and their use to improve oil content. *Curr. Opin. Biotechnol.* 87:103130. 10.1016/j.copbio.2024.103130 38579630

[B69] Kong T. Li A. Zhang L. (2025). Microalgae-based wastewater treatment: Mechanisms, strategies, and the role of biochemical composition. *J. Environ. Chem. Eng.* 13:118131. 10.1016/j.jece.2025.118131

[B70] Kubar A. A. Mehmood S. Schagerl M. Kumar S. Hu X. Zhu F. et al. (2025). Integrated hybrid Nested-bottled photobioreactor for enhanced mixing, mass transfer, and CO_2_ fixation in Arthrospira platensis raceway pond cultivation systems. *Biotechnol. Biofuels Bioprod.* 18:67. 10.1186/s13068-025-02670-1 40605037 PMC12224621

[B71] Lam T. P. Lee T.-M. Chen C.-Y. Chang J.-S. (2018). Strategies to control biological contaminants during microalgal cultivation in open ponds. *Bioresour. Technol.* 252 180–187. 10.1016/j.biortech.2017.12.088 29306613

[B72] Leal G. B. C. Santos K. N. D. Sarpal A. S. Costa J. A. V. (2026). Two-stage cultivation of the microalga *Chlorella fusca* LEB 111 using the phytohormone indole-3-acetic acid and nitrogen reduction for assessment of aviation fuel applications. *Biomass Bioenergy* 210:109046. 10.1016/j.biombioe.2026.109046

[B73] Lee J.-C. Lee B. Heo J. Kim H.-W. Lim H. (2019). Techno-economic assessment of conventional and direct-transesterification processes for microalgal biomass to biodiesel conversion. *Bioresour. Technol.* 294:122173. 10.1016/j.biortech.2019.122173 31586730

[B74] Legrand J. Artu A. Pruvost J. (2021). A review on photobioreactor design and modelling for microalgae production. *React. Chem. Eng.* 6 1134–1151. 10.1039/D0RE00450B

[B75] Li C.-T. Yelsky J. Chen Y. Zuñiga C. Eng R. Jiang L. et al. (2019). Utilizing genome-scale models to optimize nutrient supply for sustained algal growth and lipid productivity. *NPJ Syst. Biol. Appl.* 5:33. 10.1038/s41540-019-0110-7 31583115 PMC6760154

[B76] Li Y. Zhou J. Wang X. Zhang J. Fan X. Jin J. et al. (2025). Metabolic reprogramming driven by specific carbon sources modulates morphological plasticity and enhances high-value metabolite accumulation in mixotrophic *Euglena gracilis*. *Algal Res.* 91:104308. 10.1016/j.algal.2025.104308

[B77] Lim H. R. Khoo K. S. Chia W. Y. Chew K. W. Ho S.-H. Show P. L. (2022). Smart microalgae farming with internet-of-things for sustainable agriculture. *Biotechnol. Adv.* 57:107931. 10.1016/j.biotechadv.2022.107931 35202746

[B78] Liu W. Chen Y. Wang J. Liu T. (2019). Biomass productivity of *Scenedesmus dimorphus* (Chlorophyceae) was improved by using an open pond–photobioreactor hybrid system. *Eur. J. Phycol.* 54 127–134. 10.1080/09670262.2018.1519601

[B79] Lu Z. Beal C. M. Johnson Z. I. (2022). Comparative performance and technoeconomic analyses of two microalgae harvesting systems evaluated at a commercially relevant scale. *Algal Res.* 64:102667. 10.1016/j.algal.2022.102667

[B80] Lundberg S. M. Lee S.-I. (2017). “A Unified Approach to Interpreting Model Predictions,” in *Proceedings of the 31st International Conference on Neural Information Processing Systems*, (Long Beach, CA: Curran Associates Inc).

[B81] Luo Q. Bian C. Tao M. Huang Y. Zheng Y. Lv Y. et al. (2019). Genome and transcriptome sequencing of the astaxanthin-producing green microalga, *Haematococcus pluvialis*. *Genome Biol. Evol.* 11 166–173. 10.1093/gbe/evy263 30496415 PMC6330051

[B82] Maltsev Y. Kulikovskiy M. Maltseva S. (2023). Nitrogen and phosphorus stress as a tool to induce lipid production in microalgae. *Microb. Cell Fact.* 22:239. 10.1186/s12934-023-02244-6 37981666 PMC10658923

[B83] Masojídek J. Ranglová K. Lakatos G. E. Benavides A. M. S. Torzillo G. (2021). Variables governing photosynthesis and growth in microalgae mass cultures. *Processes* 9:820. 10.3390/pr9050820

[B84] Metting F. B. (1996). Biodiversity and application of microalgae. *J. Ind. Microbiol.* 17 477–489. 10.1007/BF01574779

[B85] Molina Grima E. Belarbi E. H. Acién Fernández F. G. Robles Medina A. Chisti Y. (2003). Recovery of microalgal biomass and metabolites: Process options and economics. *Biotechnol. Adv.* 20 491–515. 10.1016/S0734-9750(02)00050-2 14550018

[B86] Morais E. G. Schüler L. Pereira H. Maia I. Gangadhar K. N. Costa J. A. V. et al. (2021). “Microalgae as source of edible lipids,” in *Cultured Microalgae for the Food Industry*, eds Lafarga T. Acién G. (London: Academic Press), 147–175.

[B87] Mu D. Xin C. Zhou W. (2020). “Life Cycle Assessment and Techno-Economic Analysis of Algal Biofuel Production,” in *Microalgae Cultivation for Biofuels Production*, (London: Academic Press), 281–292.

[B88] Narayanan H. von Stosch M. Feidl F. Sokolov M. Morbidelli M. Butté A. (2023). Hybrid modeling for biopharmaceutical processes: Advantages, opportunities, and implementation. *Front. Chem. Eng.* 5:1157889. 10.3389/fceng.2023.1157889

[B89] Nayak M. Suh W. I. Chang Y. K. Lee B. (2019). Exploration of two-stage cultivation strategies using nitrogen starvation to maximize the lipid productivity in *Chlorella sp*. *HS2. Bioresour. Technol.* 276 110–118. 10.1016/j.biortech.2018.12.111 30616209

[B90] Nogales-Delgado S. (2025). Biodiesel production and life cycle assessment: Status and prospects. *Energies* 18:3338. 10.3390/en18133338

[B91] Norsker N.-H. Barbosa M. J. Vermuë M. H. Wijffels R. H. (2011). Microalgal production — A close look at the economics. *Biotechnol. Adv.* 29 24–27. 10.1016/j.biotechadv.2010.08.005 20728528

[B92] Novoveská L. Nielsen S. L. Eroldoğan O. T. Haznedaroglu B. Z. Rinkevich B. Fazi S. et al. (2023). Overview and challenges of large-scale cultivation of photosynthetic microalgae and cyanobacteria. *Marine Drugs* 21:445. 10.3390/md21080445 37623726 PMC10455696

[B93] Obidi P. O. Bayless D. J. (2025). Mechanical characterization of algal cultivation systems for enhanced mass transfer. *Algal Res.* 88:104032. 10.1016/j.algal.2025.104032

[B94] Otálora P. Guzmán J. L. Berenguel M. Acién F. G. (2023). Data-Driven pH model in raceway reactors for freshwater and wastewater cultures. *Mathematics* 11:1614. 10.3390/math11071614

[B95] Otálora P. Hoyo Á Caparroz M. González J. Guzmán J. L. Berenguel M. (2024). Classic PID-based control strategies for raceway photobioreactor biomass concentration and water level. *IFAC-PapersOnLine* 58 497–502. 10.1016/j.ifacol.2024.08.111

[B96] Ouyang M. Wang Y. Wu F. Lin Y. (2023). Continuous reactor temperature control with optimized pid parameters based on improved sparrow algorithm. *Processes* 11:1302. 10.3390/pr11051302

[B97] Pandey S. Narayanan I. Selvaraj R. Varadavenkatesan T. Vinayagam R. (2024). Biodiesel production from microalgae: A comprehensive review on influential factors, transesterification processes, and challenges. *Fuel* 367:131547. 10.1016/j.fuel.2024.131547

[B98] Pfaffinger C. E. Schöne D. Trunz S. Löwe H. Weuster-Botz D. (2016). Model-based optimization of microalgae areal productivity in flat-plate gas-lift photobioreactors. *Algal Res.* 20 153–163. 10.1016/j.algal.2016.10.002

[B99] Plouviez M. Copedo J. Ingebrigtsen R. Muir E. (2026). “Biological Contaminants in Large-Scale Microalgae Cultures,” in *Handbook of Microalgae-Based Processes and Products*, 2nd edition Edn, eds Jacob-Lopes E. Maroneze M. M. Queiroz M. I. Zepka L. Q. (London: Academic Press), 271–297.

[B100] Popa M. D. Simionov I.-A. Petrea S. M. Georgescu P.-L. Ifrim G. A. Iticescu C. (2025). Efficiency of microalgae employment in nutrient removal (Nitrogen and Phosphorous) from municipal wastewater. *Water* 17:260. 10.3390/w17020260

[B101] Porras Reyes L. Havlik I. Beutel S. (2024). Software sensors in the monitoring of microalgae cultivations. *Rev. Environ. Sci. Biotechnol.* 23 67–92. 10.1007/s11157-023-09679-8

[B102] Putt R. Singh M. Chinnasamy S. Das K. C. (2011). An efficient system for carbonation of high-rate algae pond water to enhance CO_2_ mass transfer. *Bioresour. Technol.* 102 3240–3245. 10.1016/j.biortech.2010.11.029 21123050

[B103] Ragueneau S. Cordier C. Lange A. Torres L. Moulin P. (2026). Ultrafiltration of seawater to enhance microalgae cultivation: An industrial scale study. *Sep. Purif. Technol.* 395:137875. 10.1016/j.seppur.2026.137875

[B104] Ray A. Kundu P. Ghosh A. (2023). Reconstruction of a genome-scale metabolic model of scenedesmus obliquus and its application for lipid production under three trophic modes. *ACS Synth. Biol.* 12 3463–3481. 10.1021/acssynbio.3c00516 37852251

[B105] Razzak A. S. Bahar K. Islam K. M. O. Haniffa A. K. Faruque M. O. Hossain S. M. Z. et al. (2024). Microalgae cultivation in photobioreactors: Sustainable solutions for a greener future. *Green Chem. Eng.* 5 418–439. 10.1016/j.gce.2023.10.004

[B106] Rezvani F. Rostami K. (2023). Photobioreactors for utility-scale applications: Effect of gas–liquid mass transfer coefficient and other critical parameters. *Environ. Sci. Pollut. Res.* 30 76263–76282. 10.1007/s11356-023-27644-4 37247144

[B107] Riezzo L. Kay H. Feng Y. Jing K. Zhang D. (2025). Accelerating bioprocess digital twin development by integrating hybrid modelling with transfer learning. *Chem. Eng. J.* 511:162018. 10.1016/j.cej.2025.162018

[B108] Rossi S. Carecci D. Ficara E. (2023). Thermal response analysis and compilation of cardinal temperatures for 424 strains of microalgae, cyanobacteria, diatoms and other species. *Sci. Total Environ.* 873:162275. 10.1016/j.scitotenv.2023.162275 36801411

[B109] Ryalat M. Franco E. Elmoaqet H. Almtireen N. Al-Refai G. (2024). The integration of advanced mechatronic systems into industry 4.0 for smart manufacturing. *Sustainability* 16:8504. 10.3390/su16198504

[B110] Sander K. Murthy G. S. (2010). Life cycle analysis of algae biodiesel. *Int. J. Life Cycle Assess.* 15 704–714. 10.1007/s11367-010-0194-1

[B111] Sangregorio-Soto V. Mayorga Lancheros E. Y. De La Hoz R. (2026). Recent advances in microalgae cultivation systems: Toward autonomous architecture. *Fermentation* 12:147. 10.3390/fermentation12030147

[B112] Sazandehchi P. Chegeni A. Khalilzadeh R. Bahrami A. (2025). Design and fabrication of a new image processing-based sensor for continuous non-invasive monitoring of cell density. *Iran. J. Chem. Chem. Eng.* 44 1961–1975. 10.30492/ijcce.2025.2037802.6734

[B113] Shah P. Agashe S. (2016). Review of fractional PID controller. *Mechatronics* 38 29–41. 10.1016/j.mechatronics.2016.06.005

[B114] Sharma A. K. Jaryal S. Sharma S. Dhyani A. Tewari B. S. Mahato N. (2025). Biofuels from microalgae: A review on microalgae cultivation, biodiesel production techniques and storage stability. *Processes* 13:488. 10.3390/pr13020488

[B115] Silkina A. Gayo Pelaez J. I. (2026). “Case Studies of Large-scale Cultivation in Silico Approaches for Economic Evaluations Photobioreactor Operation,” in *Innovations in the Blue Economy*, ed. Pires J. C. M. (London: Academic Press), 483–502.

[B116] Silva T. A. do Couto E. A. Assemany P. P. Costa P. A. C. Marques P. A. S. S. Paradela F. et al. (2024). Biofuel from wastewater-grown microalgae: A biorefinery approach using hydrothermal liquefaction and catalyst upgrading. *J. Environ. Manag.* 368:122091. 10.1016/j.jenvman.2024.122091 39116814

[B117] Skifa I. Chauchat N. Cocquet P.-H. Guer Y. L. (2025). Microalgae cultivation in raceway ponds: Advances, challenges, and hydrodynamic considerations. *EFB Bioeconomy J.* 5:100073. 10.1016/j.bioeco.2024.100073

[B118] Sokolov M. von Stosch M. Narayanan H. Feidl F. Butté A. (2021). Hybrid modeling — a key enabler towards realizing digital twins in biopharma? *Curr. Opin. Chem. Eng.* 34:100715. 10.1016/j.coche.2021.100715

[B119] Stine J. M. Beardslee L. A. Sathyam R. M. Bentley W. E. Ghodssi R. (2020). Electrochemical dissolved oxygen sensor-integrated platform for wireless in situ bioprocess monitoring. *Sens. Actuators B Chem.* 320:128381. 10.1016/j.snb.2020.128381

[B120] Sun X. Zhang F. Wang J. Yang Z. Huang Z. Xue R. (2025). Digital twin for smart manufacturing equipment: Modeling and applications. *Int. J. Adv. Manuf. Technol.* 137 4929–4946. 10.1007/s00170-025-15468-0

[B121] Suparmaniam U. Lam M. K. Lim J. W. Yusup S. Tan I. S. Lau S. Y. et al. (2023). Influence of environmental stress on microalgae growth and lipid profile: A systematic review. *Phytochem. Rev.* 22 879–901. 10.1007/s11101-022-09810-7

[B122] Syed T. Krujatz F. Ihadjadene Y. Mühlstädt G. Hamedi H. Mädler J. et al. (2024). A review on machine learning approaches for microalgae cultivation systems. *Comput. Biol. Med.* 172:108248. 10.1016/j.compbiomed.2024.108248 38493599

[B123] Tamayo-Castañeda R. G. Cerrillo-Rojas G. V. Ibarra-Pérez T. Ndjatchi C. Correa-Aguado H. C. (2025). A push–pull strategy to enhance biomass and lipid production in *Nannochloropsis oculata*. *Microorganisms* 13:1131. 10.3390/microorganisms13051131 40431303 PMC12114038

[B124] Tamburro J. Boyle N. R. (2025). Charting the state of GEMs in microalgae: Progress, challenges, and innovations. *Front. Plant Sci.* 16:1614397. 10.3389/fpls.2025.1614397 40584862 PMC12202545

[B125] Tao F. Zhang H. Liu A. Nee A. Y. C. (2019). Digital twin in industry: State-of-the-art. *IEEE Trans. Ind. Inform.* 15 2405–2415. 10.1109/TII.2018.2873186

[B126] Tredici M. R. Zittelli G. C. (1998). Efficiency of sunlight utilization: Tubular versus flat photobioreactors. *Biotechnol. Bioeng.* 57 187–197. 10099193 10.1002/(sici)1097-0290(19980120)57:2<187::aid-bit7>3.0.co;2-j

[B127] Uduman N. Qi Y. Danquah M. K. Forde G. M. Hoadley A. (2010). Dewatering of microalgal cultures: A major bottleneck to algae-based fuels. *JRSE* 2:012701. 10.1063/1.3294480

[B128] Vasile N. S. Cordara A. Usai G. Re A. (2021). Computational analysis of dynamic light exposure of unicellular algal cells in a flat-panel photobioreactor to support light-induced CO_2_ bioprocess development. *Front. Microbiol.* 12:639482. 10.3389/fmicb.2021.639482 33868196 PMC8049116

[B129] Wang B. Wang Z. Chen T. Zhao X. (2020). Development of novel bioreactor control systems based on smart sensors and actuators. *Front. Bioeng. Biotechnol.* 8:7. 10.3389/fbioe.2020.00007 32117906 PMC7011095

[B130] Wang C. Lan C. Q. (2018). Effects of shear stress on microalgae – A review. *Biotechnol. Adv.* 36 986–1002. 10.1016/j.biotechadv.2018.03.001 29524464

[B131] Wang M. Ye X. Bi H. Shen Z. (2024). Microalgae biofuels: Illuminating the path to a sustainable future amidst challenges and opportunities. *Biotechnol. Biofuels Bioprod.* 17:10. 10.1186/s13068-024-02461-0 38254224 PMC10804497

[B132] Wei Q. Zhao D. Wang M. Wang C. Pang F. Ma X. (2025). Enhanced lipid production in *Chlorella vulgaris* via indole-3-acetic acid salt stress in a two-stage culture for biofuels. *Biochem. Eng. J.* 221:109795. 10.1016/j.bej.2025.109795

[B133] Xia J. Liu J. Li J. Lin Y. Ge S. Qiu S. (2026). Effect of high ammonium stress on microalgae cultivation: An integrated analysis of research trends, mechanistic insights, and mitigation strategies. *Bioresour. Technol.* 452:134611. 10.1016/j.biortech.2026.134611 41980650

[B134] Xin C. Addy M. M. Zhao J. Cheng Y. Cheng S. Mu D. et al. (2016). Comprehensive techno-economic analysis of wastewater-based algal biofuel production: A case study. *Bioresour. Technol.* 211 584–593. 10.1016/j.biortech.2016.03.102 27039331

[B135] Xu P. Shao S. Qian J. Li J. Xu R. Liu J. et al. (2024). Scale-up of microalgal systems for decarbonization and bioproducts: Challenges and opportunities. *Bioresour. Technol.* 398:130528. 10.1016/j.biortech.2024.130528 38437968

[B136] Xu Y. Yu G. Wang Z. Cao W. Feng M. Wei W. et al. (2026). Integrated two-stage continuous system with pH-triggered CO_2_ delivery for sustainable algal wastewater-to-bioenergy production. *Bioresour. Technol.* 443:133900. 10.1016/j.biortech.2025.133900 41475603

[B137] Yamaoka Y. Petroutsos D. Je S. Yamano T. Li-Beisson Y. (2025). Light, CO_2_, and carbon storage in microalgae. *Curr. Opin. Plant Biol.* 84:102696. 10.1016/j.pbi.2025.102696 39983365

[B138] Yan H. Gao S. Wigmosta M. S. Coleman A. M. Sun N. Huesemann M. H. (2026). Adaptive algal cultivation enabled by a monthly biomass forecasting system. *Biotechnol. Bioeng.* 123 724–741. 10.1002/bit.70120 41351207 PMC12883906

[B139] Yang C.-T. Kristiani E. Leong Y. K. Chang J.-S. (2024). Machine learning in microalgae biotechnology for sustainable biofuel production: Advancements, applications, and prospects. *Bioresour. Technol.* 413:131549. 10.1016/j.biortech.2024.131549 39349125

[B140] Yang J. Xu M. Zhang X. Hu Q. Sommerfeld M. Chen Y. (2011). Life-cycle analysis on biodiesel production from microalgae: Water footprint and nutrients balance. *Bioresour. Technol.* 102 159–165. 10.1016/j.biortech.2010.07.017 20675125

[B141] Yu W.-L. Ansari W. Schoepp N. G. Hannon M. J. Mayfield S. P. Burkart M. D. (2011). Modifications of the metabolic pathways of lipid and triacylglycerol production in microalgae. *Microb. Cell Fact.* 10:91. 10.1186/1475-2859-10-91 22047615 PMC3234195

[B142] Yusof Z. Tong Y. W. Selvarajoo K. Parakh S. K. Foo S. C. (2025). Overcoming challenges in microalgal bioprocessing through data-driven and computational approaches. *Curr. Opin. Food Sci.* 61:101253. 10.1016/j.cofs.2024.101253

[B143] Zakova T. F. Belohlav V. Jirout T. (2025). Scalable photobioreactor monitoring: A validated approach to biofouling detection via light transmission method. *Algal Res.* 91:104314. 10.1016/j.algal.2025.104314

[B144] Zhao S. Feng W. Li J. Zhang X. Liu L. Li H. (2023). Effects of bubble cutting dynamic behaviors on microalgal growth in bubble column photobioreactor with a novel aeration device. *Front. Bioeng. Biotechnol.* 11:1225187. 10.3389/fbioe.2023.1225187 37449087 PMC10336540

[B145] Zimmermann A. W. Wunderlich J. Müller L. Buchner G. A. Marxen A. Michailos S. et al. (2020). Techno-Economic assessment guidelines for CO_2_ utilization. *Front. Energy Res.* 8:5. 10.3389/fenrg.2020.00005

